# P2RX7-MAPK1/2-SP1 axis inhibits MTOR independent HSPB1-mediated astroglial autophagy

**DOI:** 10.1038/s41419-018-0586-x

**Published:** 2018-05-10

**Authors:** Ji-Eun Kim, Ah-Reum Ko, Hye-Won Hyun, Su-Ji Min, Tae-Cheon Kang

**Affiliations:** Department of Anatomy and Neurobiology, Institute of Epilepsy Research, College of Medicine, Hallym University, Chuncheon, 24252 South Korea

## Abstract

Recently, we have reported that heat shock protein B1 (HSPB1) and purinergic receptor P2X7 (P2RX7) are involved in astroglial autophagy (clasmatodendrosis), following status epilepticus (SE). However, the underlying mechanisms of astroglial autophagy have not been completely established. In the present study, we found that the lacking of *P2rx7* led to prolonged astroglial HSPB1 induction due to impaired mitogen-activated protein kinase 1/2 (MAPK1/2)-mediated specificity protein 1 (SP1) phosphorylation, following kainic acid-induced SE. Subsequently, the upregulated HSPB1 itself evoked ER stress and exerted protein kinase AMP-activated catalytic subunit alpha 1 (PRKAA1, AMPK1)/unc-51 such as autophagy activating kinase 1 (ULK1)- and AKT serine/threonine kinase 1 (AKT1)/glycogen synthase kinase 3 beta (GSK3B)/SH3-domain GRB2-like B1 (SH3GLB1)-mediated autophagic pathways, independent of mechanistic target of rapamycin (MTOR) activity in astrocytes. These findings provide a novel purinergic suppression mechanism to link chaperone expression to autophagy in astrocytes. Therefore, we suggest that P2RX7 may play an important role in the regulation of autophagy by the fine-tuning of HSPB1 expression.

## Introduction

Astrocytes regulate extracellular ion homeostasis, brain–blood barrier, and energy metabolism that are essential for neuronal survival and synaptic function^1^. Because of reactive astrogliosis^2^, astrocytes are believed to be resistant to harmful stresses. However, a growing body of evidence demonstrates that astroglial damage occurs before or after reactive astrogliosis in epilepsy^3,4^, brain ischemia^5,6^, and Alzheimer’s disease^7^.

More than 100 years ago, Alzheimer reported irreversible astroglial injury characterized by extensive swollen vacuolized cell bodies and disintegrated/beaded processes, and Cajal termed it as “clasmatodendrosis”^8^. We have reported that status epilepticus (SE, a continuous unremitting seizure activity) evokes clasmatodendrosis, which is involved in the synchronous epileptiform discharges^9–12^. Since clasmatodendritic astrocyte shows eosinophilic cytoplasm with vacuolization and TUNEL-negativity, we had speculated that clasmatodendrosis might be coagulative necrotic events^13,14^. Unexpectedly, we have found that vacuoles in clasmatodendritic astrocytes are lysosome-associated membrane protein 1 (LAMP1)-positive, which is required for the essential activation of autophagy^9,15^. Furthermore, blockade of purinergic receptor P2X7 (P2RX7), an ATP-gated ion channel^16^, exacerbates clasmatodendrosis^17^. Thus, we have reported that clasmatodendrosis may be P2RX7-mediated astroglial autophagy^10^.

Heat shock protein B1 (HSPB1) is an inducible HSP facilitating protein folding and removal of aberrant proteins^18,19^. After kainic acid (KA)- or pilocarpine (PILO)-induced SE, HSPB1 is prominently expressed in astrocytes, not in neurons^20,21^. Thus, HSPB1 is a sensitive and reliable representative marker of the early astroglial energy-consuming events^21,22^. Interestingly, impaired clearance of HSPB1 reduces astroglial viability^23^. Indeed, prolonged HSPB1 induction results in endoplasmic reticulum (ER) stress, and subsequently switches on astroglial autophagy^12^. However, it is still unknown how the sustained HSPB1 expression turns on astroglial autophagy and whether P2RX7 is involved in this autophagic pathway. In the present study, therefore, we investigate the role of P2RX7 in HSPB1-mediated astroglial autophagy and the downstream signaling pathways in this process.

Here we demonstrate for the first time that deletion or blockade of P2RX7 results in the prolonged HSPB1 induction by impaired mitogen-activated protein kinase 1/2 (MAPK1/2)-mediated Sp1 transcription factor (SP1) phosphorylation in astrocytes following KA injection. In turn, sustained HSPB1 expression activates eukaryotic translation initiation factor 2 subunit alpha (EIF2S1)/activating transcription factor 4 (ATF4) pathway, which accelerates protein kinase AMP-activated catalytic subunit alpha 1 (PRKAA1, AMPK1)/unc-51 like autophagy activating kinase 1 (ULK1)-mediated autophagy. Prolonged HSPB1 induction also exerts AKT serine/threonine kinase 1 (AKT1)/glycogen synthase kinase 3 beta (GSK3B)/SH3-domain GRB2-like B1 (SH3GLB1)-mediated autophagic pathway, independent of phosphatidylinositol 3-kinase (PIK3) activity. Mechanistic target of rapamycin (MTOR) is not involved in these two distinct pathways. Therefore, we suggest that P2RX7 may be one of the fundamental regulators for astroglial autophagy via the fine-tuning of HSPB1 expression.

## Results

### *P2rx7* deletion evokes prolonged astroglial HSPB1 induction and astroglial autophagy without change in seizure susceptibility in response to KA

First, we evaluated the seizure susceptibilities of *P2rx7*^*+/+*^ wild-type (WT) and *P2rx7*^*−/−*^ (KO) mice in responses to PILO and KA, because HSPB1 induction correlated to the degree of seizure activity^21^. Consistent with our previous study^16^, KO mice showed seizure activity in response to subconvulsive dose (200 mg/kg) of PILO to WT animals (Supplementary Figure 1A-B). A single dose of 350 mg/kg-PILO injection effectively induced SE in WT mice, while it was lethal to KO mice (Supplementary Figure 1A-B). Due to the higher dose of PILO, the total power of EEG during SE in WT mice was larger than that observed in KO mice (*p* < 0.05; Supplementary Figure 1B). However, astroglial HSPB1 and LAMP1 expressions in KO mice were higher than those in the WT mice 7 days after SE (*p* < 0.05; Supplementary Figure 1C-D). These findings indicate that *P2rx7* deletion may promote astroglial HSPB1 induction and autophagy, irrelevant to PILO-induced seizure severity.

**Fig. 1:**
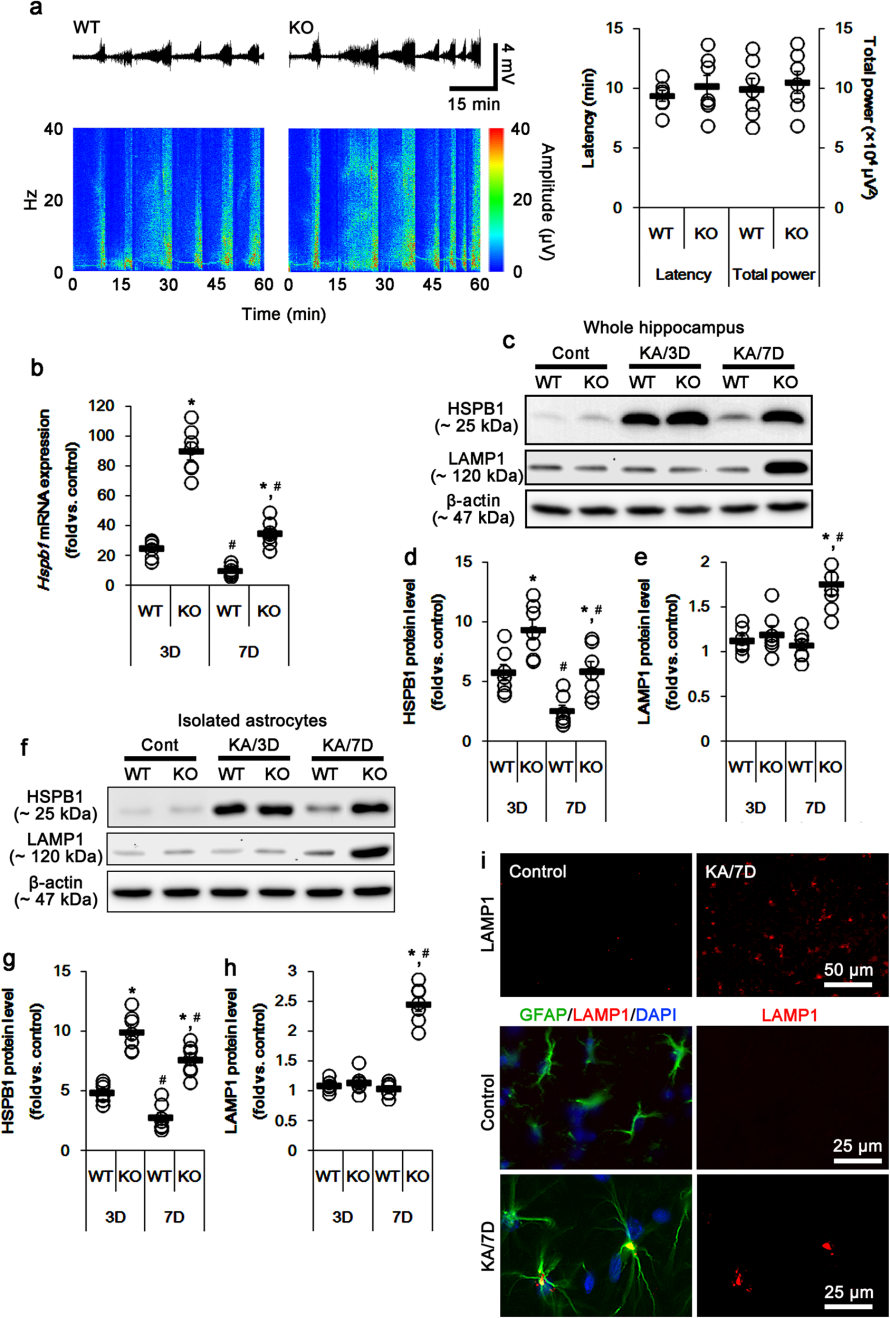
Effect of *P2rx7* deletion on HSPB1-mediated autophagy in response to KA. **a** Effect of *P2rx7* deletion on seizure susceptibility in response to KA. *P2rx7* deletion does not affect the seizure susceptibility in response to KA. (Left panel) Representative EEG traces and frequency-power spectral temporal maps. (Right panel) Quantification of SE induction, latency, and total EEG power in response to KA. Open circles indicate each individual value. Horizontal bars indicate mean value. Error bars indicate SEM (*n* = 7, respectively). **b** Effect of *P2rx7* deletion on *Hspb1* mRNA expression in response to KA. *P2rx7* KO mice show the higher *Hspb1* mRNA expression following KA injection, as compared to WT animals (*,^*#*^*p* < 0.05 vs. WT mice and 3 days after SE, respectively; *n* = 7, respectively). **c**–**e** Effect of *P2rx7* deletion on SE-induced HSPB1 and LAMP1 expressions in the whole hippocampus. As compared to WT mice, both HSPB1 and LAMP1 expression levels are higher in KO mice. **c** Representative western blot of HSPB1 and LAMP1. Cont control animals, KA/3D 3 days post KA injected in animals, KA/7D 7 days post KA injected in animals. **d**–**e** Quantifications of HSPB1 and LAMP1 expressions following KA injection (*,^*#*^*p* < 0.05 vs. WT mice and 3 days after SE, respectively; *n* = 7, respectively). **f**–**h** Effect of *P2rx7* deletion on SE-induced HSPB1 and LAMP1 protein expressions in purified astrocytes. As compared to WT astrocytes, both HSPB1 and LAMP1 expression levels are higher in *P2rx7-*deleted astrocytes. **f** Representative western blot of HSPB1 and LAMP1. **g**–**h** Quantifications of HSPB1 and LAMP1 expressions following KA injection (*,^*#*^*p* < 0.05 vs. WT mice and 3 days after SE, respectively; *n* = 7, respectively). **i** Representative photos demonstrating astroglial LAMP1 expression in the KO mouse hippocampus 7 days after KA injection. *P2rx7* deletion increases LAMP1 expression, as compared to WT animals (see also supplementary Figure 1)

Consistent with our previous report^16^, the present data demonstrates no difference in KA-induced seizure activity between WT and KO mice (Fig. 1a). Three days after SE, both WT and KO mice showed the upregulation of *Hspb1* mRNA expression in the hippocampus. However, *Hspb1* mRNA level in the KO mice was higher than that in WT mice (*p* < 0.05; Fig. 1b). Seven days after SE, *Hspb1* mRNA expression was reduced in both the groups (*p* < 0.05 vs. 3 days after SE, respectively; Fig. 1b), but its expression was still higher in the KO mice than in the WT mice (*p* < 0.05; Fig. 1b). HSPB1 protein level in KO mice was also higher than that observed in WT mice 3 and 7 days after SE (*p* < 0.05, respectively; Fig. 1c–e). Prolonged HSPB1 expression was observed in astrocytes, not in neurons, microglia, or oligodendroglia (Supplementary Figures 2A, 3A-B, 4A-C). *P2rx7*-deleted astrocytes showed the prolonged HSPB1 induction after KA injection, as compared to WT astrocytes (*p* < 0.05; Fig. 1f–h). Together with the data obtained from PILO injection, our findings suggest that *P2rx7* deletion may result in prolonged HSPB1 induction, independent of seizure activity.

LAMP1 is important for the autophago-lysosomal pathway^24–26^. Thus, we investigated whether *P2rx7* deletion affects LAMP1 expression in the hippocampus following KA injection. Seven days after KA injection, LAMP1 expression was upregulated in KO mice, not in WT mice (*p* < 0.05, Fig. 1c–e). *P2rx7*-deleted astrocytes also showed the increased LAMP1 expression after KA injection (*p* < 0.05 vs. WT astrocytes; Fig. 1f–h). KA upregulated astroglial LAMP1 expression in KO mice was more than WT mice (Fig. 1i and Supplementary Figures 2B, 3, 4). These findings indicated that *P2rx7* deletion may evoke the persistent HSPB1 induction, which exerts astroglial autophagy without changed seizure susceptibility in response to KA.

To confirm the role of P2RX7 in HSPB1 and LAMP1 expressions, we applied P2RX7 agonist (BzATP) and antagonists (OxATP and A740003) to WT mice. Seven days after KA injection, BzATP reduced HSPB1 induction, while P2RX7 antagonists elevated HSPB1 and LAMP1 expressions (*p* < 0.05 vs. vehicle, respectively; Supplementary Figure 5A-B). Furthermore, BzATP induced astroglial apoptosis 7 days after KA injection (*p* < 0.05 vs. vehicle; Supplementary Figure 5C-D), while the vehicle and A740003 did not (Supplementary Figure 5C-D). These findings suggest that P2RX7 inhibition may accelerate HSPB1 induction and autophagy, while P2RX7 activation may induce apoptosis in astrocytes accompanied by reduction in HSPB1 expression.

### Prolonged HSPB1 induction promotes ER stress-mediated autophagy in *P2rx7-*deleted astrocytes

**Fig. 2:**
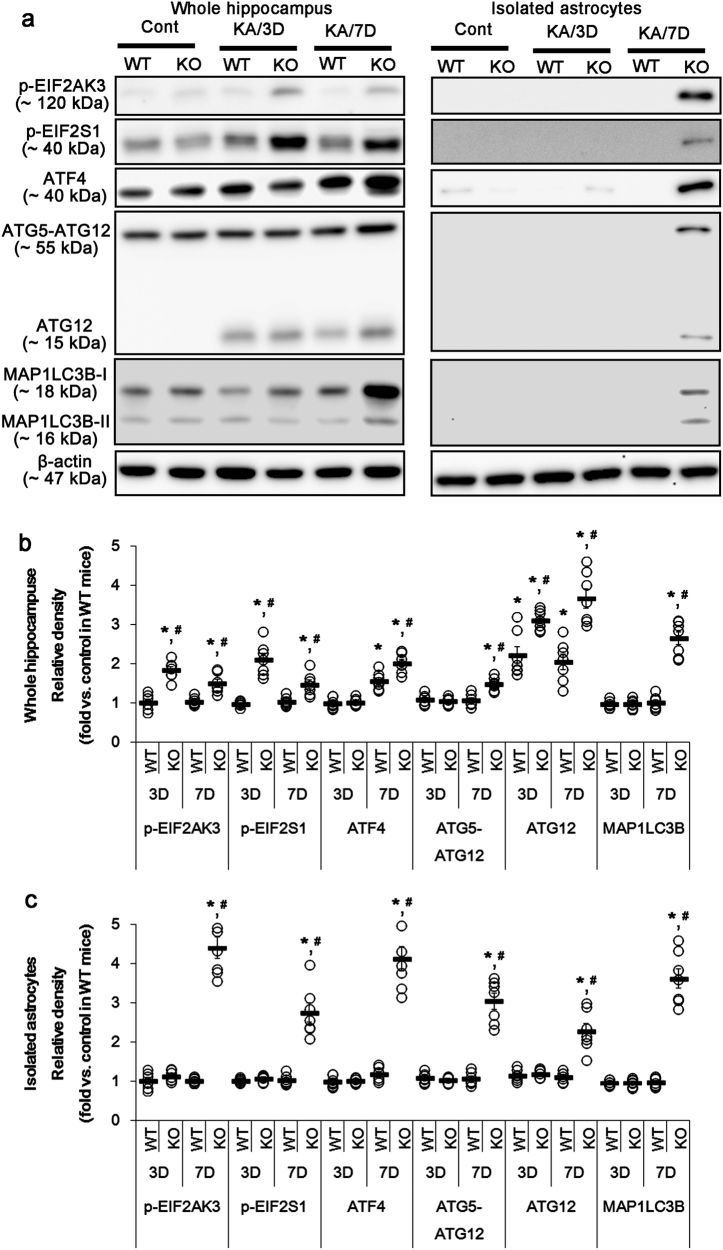
KA-induced ER stress and autophagy. **a** Representative western blot of ER stress- and autophagy-related molecules in the whole hippocampus (Left panels) and isolated astrocytes (Right panels). *P2rx7* deletion induces ER stress and autophagy following KA injection. Cont control animals, KA/3D 3 days post KA injected in animals, KA/7D 7 days post KA injected in animals. **b**–**c** Quantifications of ER stress-related molecules in the whole hippocampus (**b**) and isolated astrocytes (**c**) following KA injection. Open circles indicate each individual value. Horizontal bars indicate mean value. Error bars indicate SEM (*,^*#*^*p* < 0.05 vs. control and WT mice, respectively; *n* = 7, respectively)

**Fig. 3:**
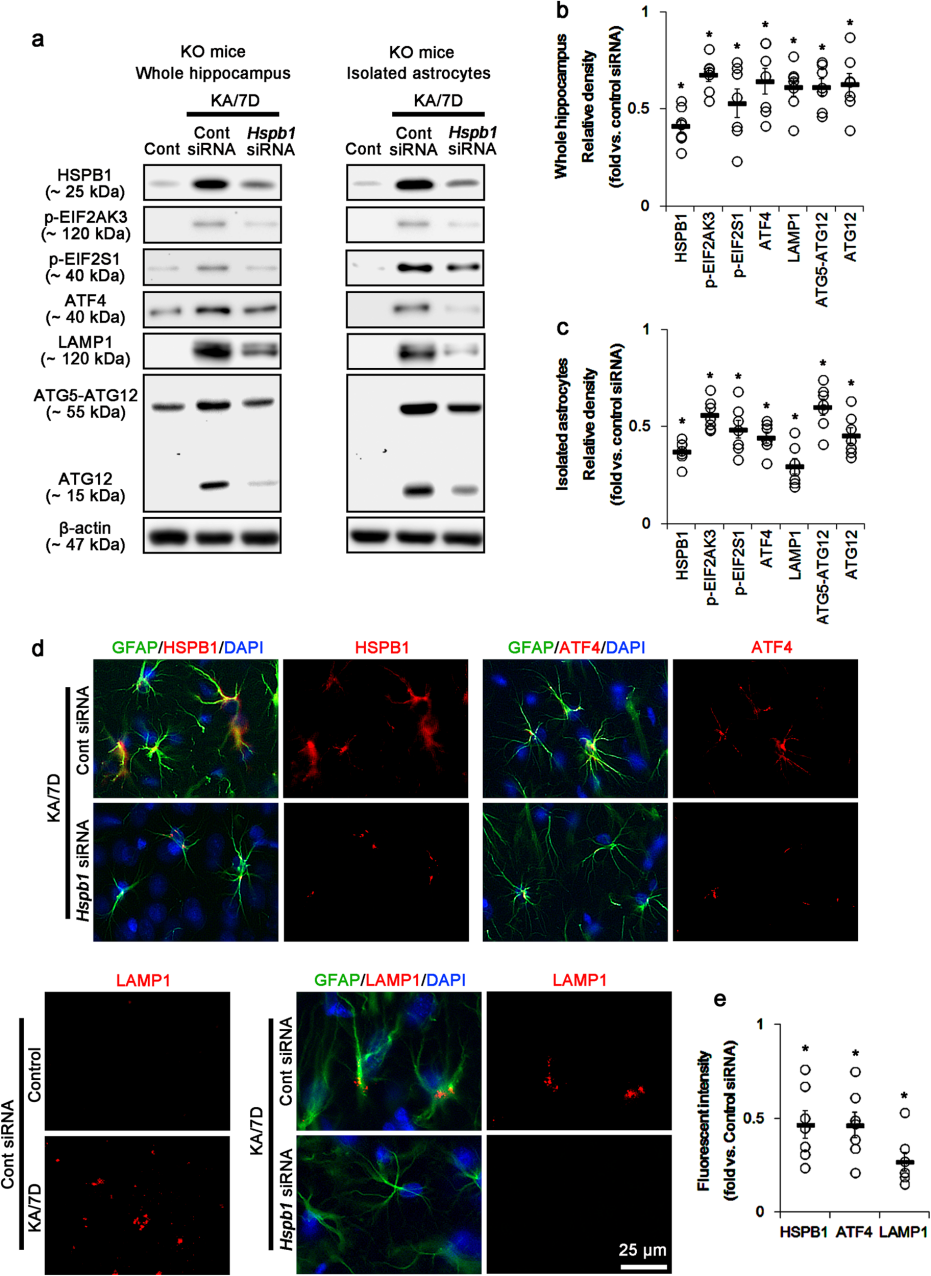
Effect of *Hspb1* knockdown on HSPB1-mediated astroglial ER stress and autophagy in the whole KO hippocampus and *P2rx7-*deleted astrocytes. *Hspb1* siRNA abrogates ER stress and autophagy in the whole KO hippocampus and *P2rx7-*deleted astrocytes. **a** Representative western blot of ER stress- and autophagy-related molecules in the whole hippocampus (Left panels) and isolated astrocytes (Right panels) 7 days after KA injection (KA/7D). **b**–**c** Quantifications of ER stress-related molecules in the whole hippocampus (**b**) and isolated astrocytes (**c**) following KA injection. Open circles indicate each individual value. Horizontal bars indicate the mean value. Error bars indicate SEM (*p < 0.05 vs. control siRNA; *n* = 7, respectively). **d** Representative photos demonstrating astroglial HSPB1, ATF4, and LAMP1 expression in the KO mouse hippocampus 7 days after KA injection. **e** Quantifications of effect of *Hspb1* siRNA on astroglial ER stress and autophagy (*p < 0.05 vs. control siRNA; *n* = 7, respectively)

ER stress activates versatile ER sensor proteins including eukaryotic translation initiation factor 2 alpha kinase 3 (EIF2AK3), endoplasmic reticulum to nucleus signaling 1 (ERN1), and activating transcription factor 6 (ATF6)^27^. Furthermore, EIF2AK3/EIF2S1/ATF4 pathway mediates autophagy through ATF4-driven transcriptional regulation of *Atg* genes including autophagy-related 12 (*Atg12*) and microtubule-associated proteins 1A/1B light chain 3B (MAP1LC3B)^28–30^. In the present study, *P2rx7* deletion significantly increases phospho (p)-EIF2AK3, p-EIF2S1, and ATF4 levels in the hippocampus after KA injection (*p* < 0.05 vs. WT mice, respectively; Fig. 2a–b). *P2rx7* deletion also elevates the expression levels of ATG12, ATG5-ATG12, and MAP1LC3B 7 days after KA treatment (*p* < 0.05 vs. WT mice, respectively; Fig. 2a–b). *P2rx7-*deleted astrocytes also revealed the upregulations of p-EIF2AK3, p-EIF2S1, ATG12, ATG5-ATG12, and MAP1LC3B expressions 7 days after KA treatment (*p* < 0.05 vs. WT astrocytes, respectively; Fig. 2a, c). However, *P2rx7* deletion did not affect p-ERN1, DNA damage inducible transcript 3, and ATF6 levels (Supplementary Figure 6A-B). *Hspb1* siRNA effectively abolished the upregulations of HSPB1, pEIF2AK3, p-EIF2S1, ATF4, ATG12, ATG5-ATG12, and LAMP1 expression in the KO mouse hippocampus and *P2rx7*-deleted astrocytes 7 days after SE (*p* < 0.05 vs. control siRNA; Fig. 3a–c). Immunostaining revealed that *Hsp1B* siRNA attenuated the upregulations of HSPB1, ATF4, and LAMP1 expression in astrocytes within the KO mouse hippocampus after KA injection (*p* < 0.05 vs. control siRNA, respectively; Fig. 3d, e). Thus, our findings suggest that *P2rx7* deletion may result in HSPB1-mediated ER stress and subsequently turn on astroglial autophagy via EIF2AK3/EIF2S1/ATF4 signaling pathway.

### *P2rx7* deletion promotes ULK1-mediated autophagy independent of MTOR

**Fig. 4:**
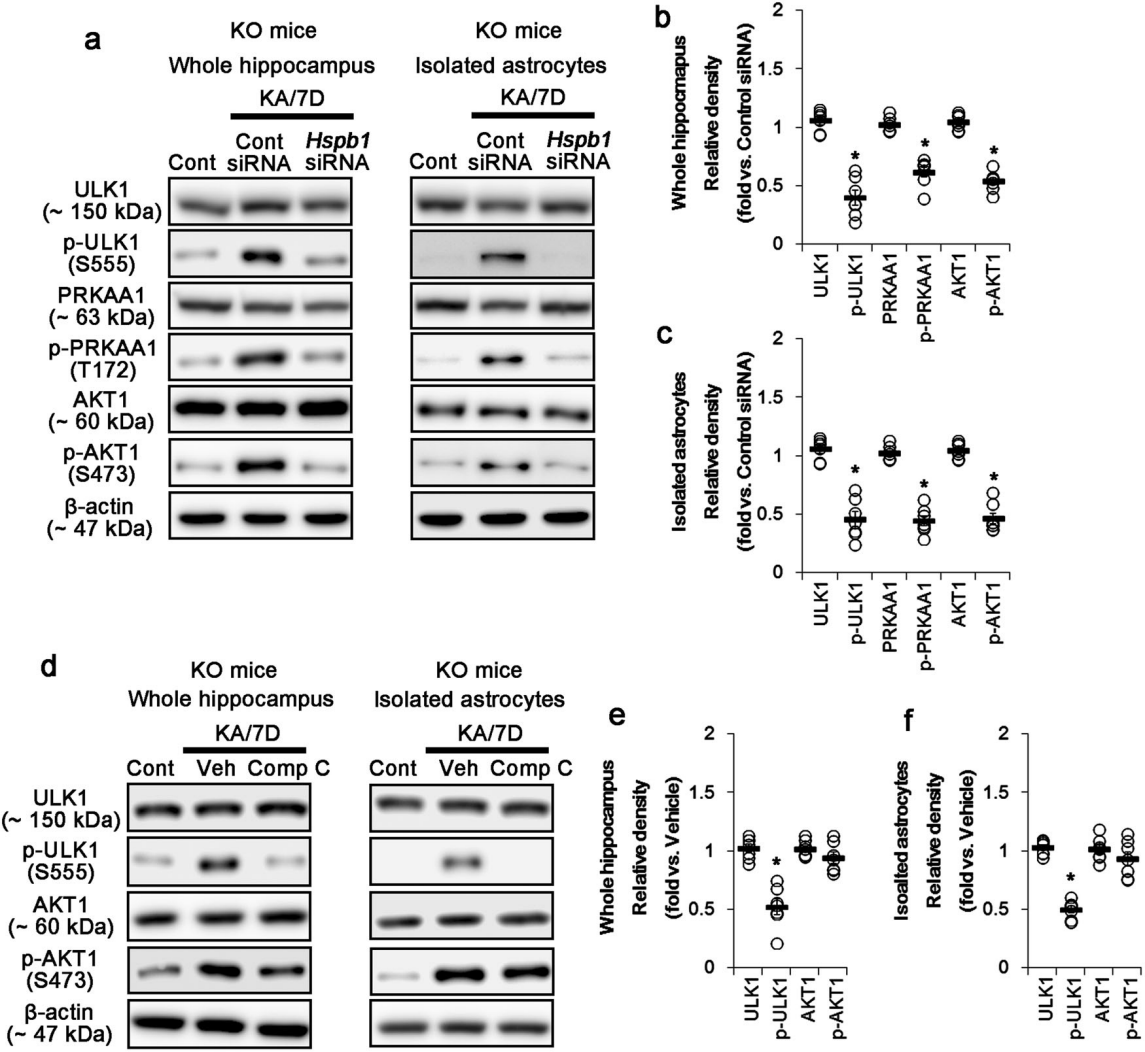
Effect of *Hspb1* knockdown and Comp C on ULK1-meidated astroglial autophagy in the whole KO hippocampus and *P2rx7-*deleted astrocytes following KA injection. **a** Representative western blot for the effect of *Hspb1* knockdown on ULK1-related molecules in the whole hippocampus (Left panels) and isolated astrocytes (Right panels). *Hspb1* siRNA decreases ULK1-S555 and PRKAA1-T173 phosphorylations without altering their expressions 7 days after KA injection (KA/7D). **b**–**c** Quantifications of effect of *Hspb1* knockdown on ULK1-related molecules in the whole hippocampus (**b**) and isolated astrocytes (**c**). Open circles indicate each individual value. Horizontal bars indicate mean value. Error bars indicate SEM (*p < 0.05 vs. control siRNA; *n* = 7, respectively). **d** Representative western blot for the effect of PRKAA1 inhibition on ULK1-related molecules in the whole hippocampus (Left panels) and isolated astrocytes (Right panels). As compared to vehicle (Veh), Comp C inhibits ULK1-S555 phosphorylation without altered its expression 7 days after KA injection. **e**–**f** Quantifications of effect of *Hspb1* knockdown on ULK1-related molecules in the whole hippocampus (**e**) and isolated astrocytes (**f**) (*p < 0.05 vs. control siRNA; *n* = 7, respectively)

ULK1 is one of the core machinery of initiation of autophagy, which is reversely regulated by MTOR and PRKAA1 kinase: PRKAA1 activates autophagy via ULK1-S555 site phosphorylation, while MTOR inhibits autophagy by reducing ULK1 kinase activity through the ULK1-S757 site phosphorylation^31^. In the present study, KA does not affect ULK1 and PRKAA1 expressions and their phosphorylations in the WT mouse hippocampus until 7 days after KA treatment (Supplementary Figure 7A-C). However, *P2rx7* deletion increases phosphorylation levels of PRKAA1 and ULK1-S555 (not ULK1-S757) 7 days after KA treatment (*p* < 0.05 vs. WT mice; Supplementary Figure 7A-E). These findings indicate that *P2rx7* deletion may increase PRKAA1-mediated ULK1 activity following KA injection. Although, *Hspb1* knockdown did not affect expression/phosphorylation levels of ULK1 and PRKAA1 in the control KO animals (Supplementary Figure 8A-C), it significantly attenuated ULK1-S555 phosphorylation, accompanied by abolished PRKAA1 activity in the KO mouse hippocampus and *P2rx7-*deleted astrocytes 7 days after KA injection (*p* < 0.05 vs. control siRNA, respectively; Fig. 4a–c). Compound c (Comp C, a PRKAA1 inhibitor) also diminished ULK1-S555 phosphorylation in the KO mouse hippocampus and *P2rx7-*deleted astrocytes after KA injection (*p* < 0.05 vs. vehicle, respectively; Fig. 4d–f). Both *Hspb1* siRNA and Comp C prevented SE-induced ULK1-S555 phosphorylation in astrocytes (Supplementary Figures 9 A-C). These findings suggest that prolonged HSPB1 expression by *P2rx7* deletion may accelerate PRKAA1/ULK1-mediated astroglial autophagy following KA injection.

Next, we examined the activities of MTOR and MTOR regulators such as PIK3, phosphatase and tensin homolog (PTEN)^32^. MTOR expression and MTOR-S2448 (not-S2481) phosphorylation were similarly reduced in the hippocampi of both WT and KO mice 3 days after KA injection (*p* < 0.05 vs. control, respectively; Supplementary Figure 10A-C). Seven days after SE, MTOR expression, and MTOR-S2448 phosphorylation recovered to control levels in both WT and KO mice. Expression/phosphorylation level of ribosomal protein S6 kinase beta-1 (RPS6KB1, a MTOR substrate) and PTEN were unaltered in both WT and KO mice (Supplementary Figure 10A-C). However, PIK3-Y458 phosphorylation level was similarly reduced in both WT and KO mice (*p* < 0.05 vs. control; Supplementary Figures 10A,C). Since PIK3 inhibits MTOR activity by AKT1/AKT1 substrate 1 (AKT1S1)^33^, we verified whether the reduced PIK3 phosphorylation influences AKT1 and AKT1S1 activities. In the present study, KA does not affect AKT1 and AKT1S1 expressions in the hippocampi of both WT and KO mice (Supplementary Figures 7A-E and 10A-C). In KO mouse, hippocampus, and *P2rx7*-deleted astrocytes, however, KA increased AKT1-S473 (not-T308 and -T450) phosphorylation (*p* < 0.05 vs. WT mice and WT astrocytes, respectively; Supplementary Figures 7A-E and 10A-C). These findings suggest that *P2rx7* deletion may facilitate PRKAA1/ULK1-mediated astroglial autophagy, independent of MTOR.

### *P2rx7* deletion induces SH3GLB1-mediated autophagy by the enhanced AKT1-S473 phosphorylation

**Fig. 5:**
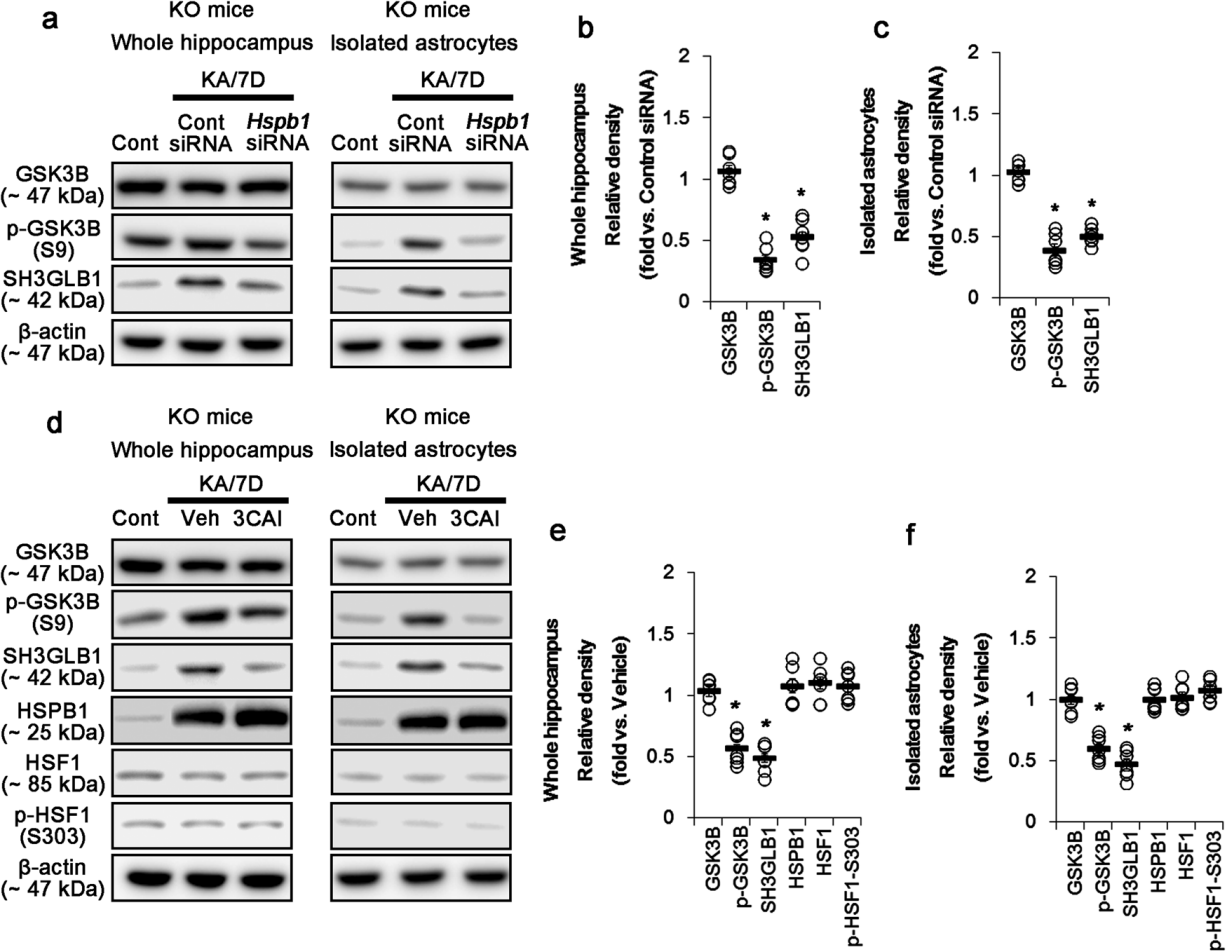
Effect of *Hspb1* knockdown and 3CAI on AKT1-GSK3B/SH3GLB1-mediated astroglial autophagy in the whole KO hippocampus and *P2rx7-*deleted astrocytes following KA injection. **a** Representative western blot for the effect of *Hspb1* knockdown on GSK3B and SH3GLB1 expression and their phosphorylations. *Hspb1* siRNA decreases GSK3B-S3 phosphorylation and SH3GLB1 expression in KO mice 7 days after KA injection (KA/7D). **b**–**c** Quantifications of effect of *Hspb1* knockdown on GSK3B phosphorylation and SH3GLB1 expression in the whole hippocampus (**b**) and isolated astrocytes (**c**). Open circles indicate each individual value. Horizontal bars indicate mean value. Error bars indicate SEM (*p < 0.05 vs. control siRNA; *n* = 7, respectively). **d** Representative western blot of the effect of AKT1 inhibition on GSK3B, SH3GLB1, HSPB1, and HSF1 expression and their phosphorylations. As compared to vehicle (Veh), 3CAI inhibits GSK3B-S3 phosphorylation and SH3GLB1 expression in KO mice without altered HSPB1 expression and HSF1 phosphorylation 7 days after KA injection. **e**–**f** Quantifications of effect of 3CAI on GSK3B, SH3GLB1, HSPB1, and HSF1 expression and their phosphorylations in the whole hippocampus (**e**) and isolated astrocytes (**f**) (*p < 0.05 vs. vehicle; *n* = 7, respectively)

Unexpectedly, we found that *P2rx7* deletion increased PIK3-independent AKT1-S473 phosphorylation after KA treatment (Supplementary Figures 7A-E and 10A-C). HSPB1 increases AKT1-S473 phosphorylation, and ATK1 exerts MTOR independent autophagy by regulating GSK3B-mediated SH3GLB1 accumulation^34–36^. Therefore, it is likely that prolonged HSPB1 induction by *P2rx7* deletion will also evoke astroglial autophagy by AKT1/GSK3B/SH3GLB1 cascade. In the present study, *P2rx7* deletion increases GSK3B-S9 phosphorylation (indicating the reduced GSK3B activity) and SH3GLB1 expression in the hippocampus and purified astrocytes after KA treatment (*p* < 0.05 vs. WT mice, respectively; Supplementary Figure 10A-E). *Hspb1* knockdown and 3-chloroacetyl indole (3CAI, an AKT1 inhibitor) mitigate the increases in GSK3B phosphorylation and SH3GLB1 expression in the KO mouse hippocampus and *P2rx7*-deleted astrocytes after KA injection (*p* < 0.05 vs. control siRNA and vehicle, respectively; Fig. 5a–f; Supplementary Figure 8A-C; Supplementary Figure 12A-C). Therefore, our findings indicate that prolonged HSPB1 induction by *P2rx7* deletion may also accelerate astroglial autophagy via AKT1/GSK3B/SH3GLB1 pathway.

### *P2rx7* deletion induces prolonged HSPB1 expression by reduction in MAPK1/2-mediated SP1 phosphorylation

The remaining question is how *P2rx7* deletion results in prolonged HSPB1 expression in astrocytes. AKT1 increases HSPB1 transactivation by inhibiting GSK3B-mediated heat shock transcription factor 1 (HSF1) phosphorylation^37^. Therefore, it is likely that AKT1-S473 phosphorylation will be involved in the positive feedback loop for the prolonged HSPB1 induction. However, there is no difference in HSF1 expression/phosphorylation in the hippocampus between WT and KO mice following KA injection (Fig. 5d–f and Supplementary Figure 11F-G). Furthermore, 3CAI does not alter HSPB1 expression and HSF1 phosphorylation in KO mice after KA treatment (Fig. 5d–f). These data indicate that *P2rx7* deletion may not induce HSF1-mediated HSPB1 transactivation following KA injection.

**Fig. 6:**
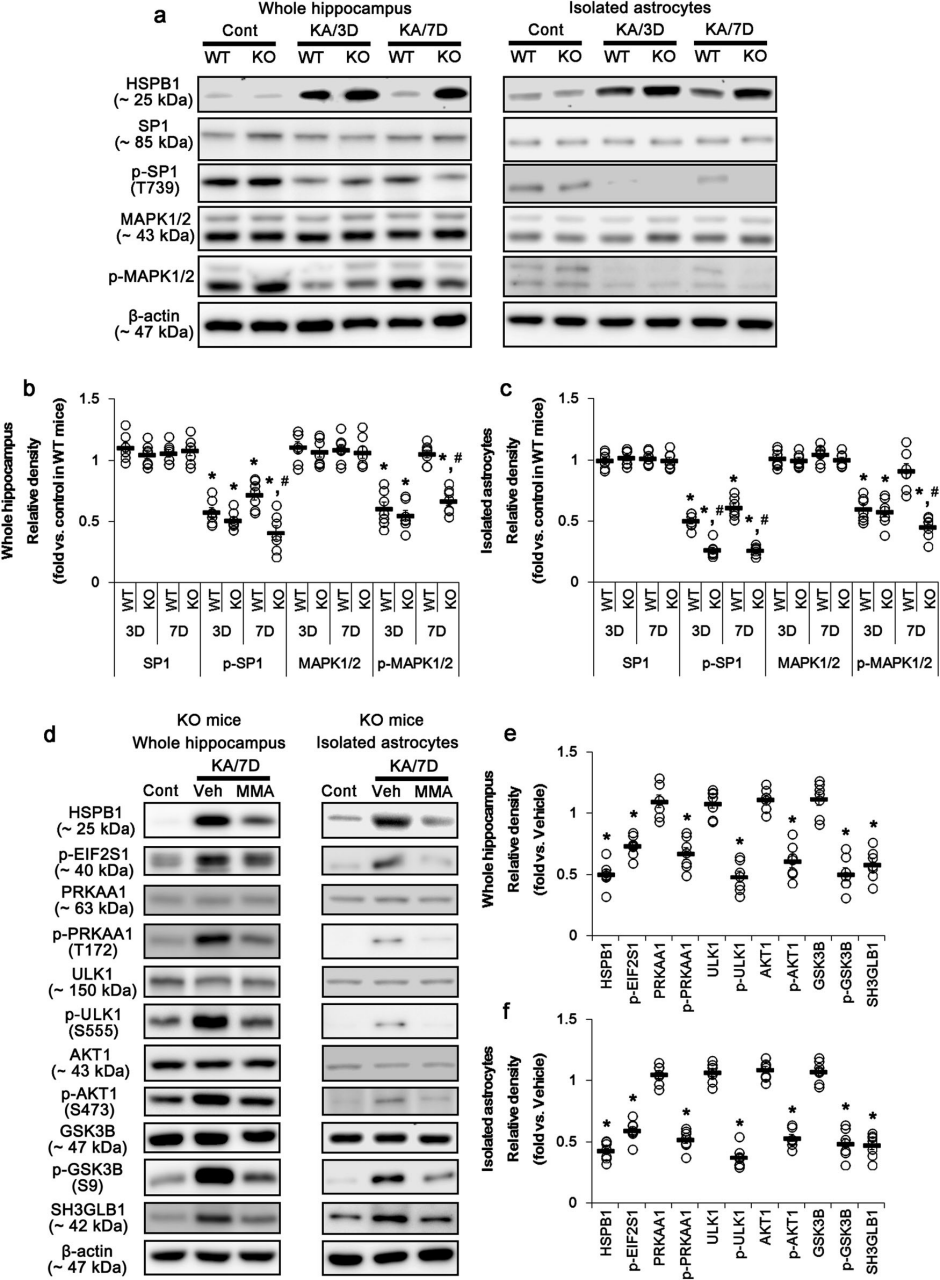
MAPK1/2-SP1-HSPB1-mediated astroglial autophagy in KO mice following KA injection. **a** Representative western blot of MAPK1/2 and SP1 expression, and their phosphorylations in the whole hippocampus (Left panels) and isolated astrocytes (Right panels). As compared to WT mice, KA injection reduced MAPK1/2 and SP1-T739 phosphorylations in KO mice without altered their expressions 7 days after SE. Cont control animals, KA/3D 3 days post KA injected in animals, KA/7D 7 days post KA injected in animals. **b**–**c** Quantifications of MAPK1/2 and SP1 expressions, and their phosphorylations in the whole hippocampus (**b**) and isolated astrocytes (**c**). Open circles indicate each individual value. Horizontal bars indicate mean value. Error bars indicate SEM (*,^*#*^*p* < 0.05 vs. control and WT mice, respectively; *n* = 7, respectively). **d** Representative western blot of the effect of SP1 inhibition on PRKAA1- and AKT1-mediated autophagy in the whole KO mouse hippocampus (Left panels) and *P2rx7*-deleted astrocytes (Right panels). As compared to vehicle (Veh), Mithramycin A (MMA) effectively inhibits PRKAA1- and AKT1-mediated signaling pathways following KA injection. **e**–**f** Quantifications of the effect of MMA on PRKAA1- and AKT1-mediated signaling pathways in the whole hippocampus (**e**) and isolated astrocytes (**f**). (*p < 0.05 vs. vehicle; *n* = 7, respectively)

**Fig. 7:**
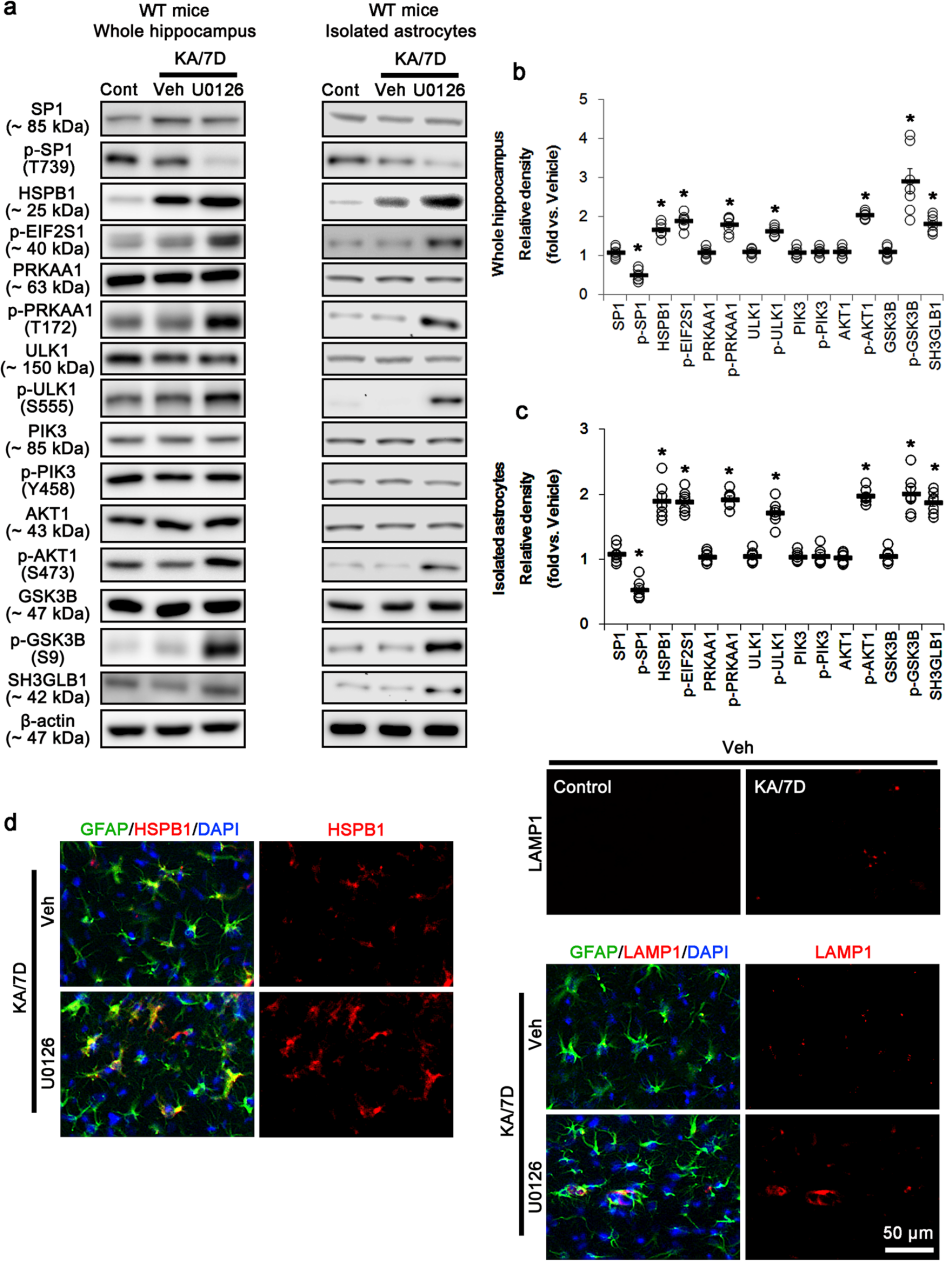
Effect of MAPK1/2 inhibition on HSPB1 expression in the whole WT hippocampus and WT astrocytes following KA injection. **a** Representative western blot of autophagy-related molecules in the whole hippocampus (Left panels) and isolated astrocytes (Right panels). As compared to vehicle (Veh), U0126 leads to HSPB1-mediated astroglial autophagy in WT mice 7 days after SE (KA/7D). **b**–**c** Quantifications of effect of U0126 on HSPB1-mediated astroglial autophagy in the whole hippocampus (**b**) and isolated astrocytes (**c**). Open circles indicate each individual value. Horizontal bars indicate mean value. Error bars indicate SEM (*p < 0.05 vs. vehicle, respectively; *n* = 7, respectively). **d** Representative photos demonstrating the effect of U0126 on HSPB1 and LAMP1 expression in WT mice 7 days after KA injection. U0126 increases both HSPB1 and LAMP1 expression in astrocytes

HSPB1 expression relies on SP1 binding to its promoter region^38,39^. MAPK1/2-mediated SP1-T739 phosphorylation inhibits HSPB1 transactivation by reducing the SP1 DNA-binding ability and regulates optimal HSP responses^40–43^. Interestingly, P2RX7 activation increases MAPK1/2 phosphorylation^44^, which inhibits HSPB1 expression^45^. These reports provide the possibility that P2RX7 may play a role in the inhibition of HSPB1 expression via MAPK1/2-mediated SP1-T739 phosphorylation. In the present study, KA significantly decreases SP1-T739 phosphorylation within the hippocampi of both WT and KO mice 3 and 7 days after KA injection (*p* < 0.05 vs. control, respectively; Fig. 6a–b). Seven days after SE, SP1 phosphorylation was reduced in the KO mice more than in WT mice (*p* < 0.05; Fig. 6a–b). Similarly, SP1 phosphorylation level in *P2rx7-*deleted astrocytes was lower than that observed in WT astrocytes (*p* < 0.05; Fig. 6a, c). Mithramycin A (MMA, a SP1 DNA-binding transcriptional inhibitor) effectively inhibited the prolonged HSPB1 induction, EIF2S1 phosphorylation, PRKAA1/ULK1- and AKT1/GSK3B/SH3GLB1 signaling pathways in the KO mouse hippocampus and *P2rx7-*deleted astrocytes after KA treatment (*p* < 0.05 vs. vehicle, respectively; Fig. 6d–f). MMA obviously prevented the upregulation of HSPB1 and LAMP1 expression in astrocytes within the KO mouse hippocampus after KA treatment (Supplementary Figure 13A-B). Similar to SP1-T739 phosphorylation, MAPK1/2 phosphorylation level was reduced in the WT mouse hippocampus and WT astrocytes 3 days after KA treatment (*p* < 0.05 vs. control, respectively; Fig. 6a–c), and recovered to the control level 7 days after SE (Fig. 6a–c), while *P2rx7* deletion did not (*p* < 0.05 vs. WT mice and WT astrocytes, respectively; Fig. 6a–c). These findings indicate that *P2rx7* deletion may evoke prolonged HSPB1 induction due to the impaired MAPK1/2-mediated SP1-T739 phosphorylation. Indeed, U0126 (a MAPK1/2 inhibitor) led to prolonged HSPB1 induction, exerted EIF2S1 phosphorylation, and activated PRKAA1/ULK1- and AKT1/GSK3B/SH3GLB1-mediated astroglial autophagy in the WT mouse hippocampus and astrocytes, accompanied by reduced SP1-T739 phosphorylation after KA injection (*p* < 0.05 vs. vehicle, respectively; Fig. 7a–d). Therefore, our findings suggest that *P2rx7* deletion may provoke HSPB1-induced astroglial autophagy via impaired MAPK1/2-mediated SP1-T739 phosphorylation.

## Discussion

**Fig. 8:**
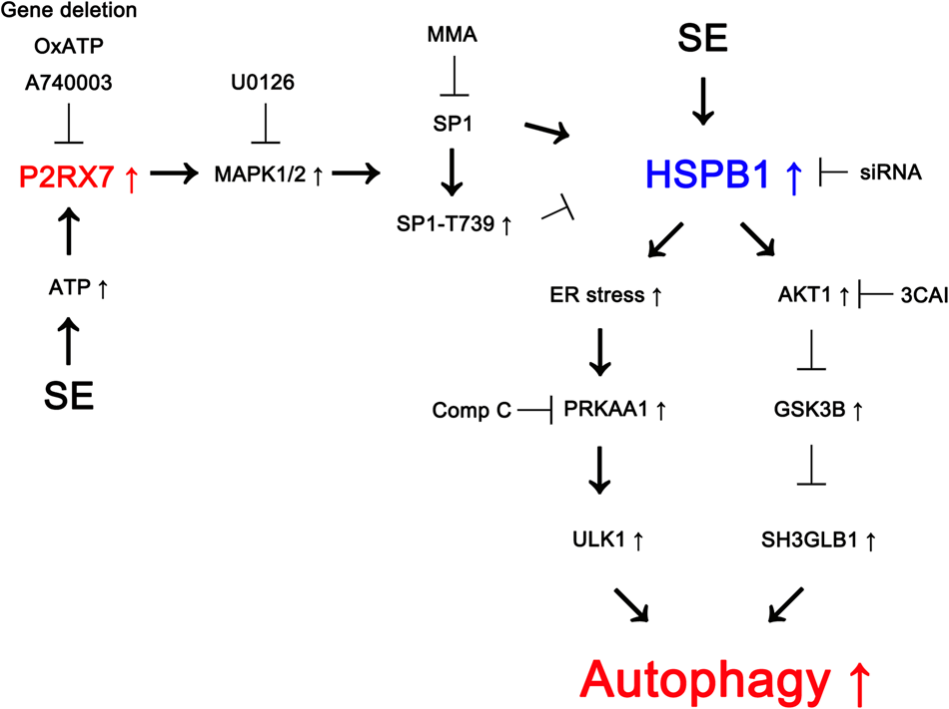
Scheme of inhibitory role of P2RX7 in astroglial autophagy induced by KA injection. After SE, P2RX7 activation inhibited HSPB1 transactivation via MAPK1/2-mediated SP1-T739 phosphorylation. However, *P2rx7* deletion or its antagonists impaired this inhibitory signaling pathway, which led to sustained HSPB1 expression and ER stress. Subsequently, ER stress activated PRKAA1/ULK1-mediated astroglial autophagy. Sustained HSPB1 expression also exerted AKT1/GSK3B-mediated SH3GLB1 accumulation, which triggered astroglial autophagy. Thus, P2RX7 regulated ER stress and autophagy by the fine-tuning of HSPB1 expression in astrocytes

The major novel finding in the present study is that *P2rx7* deletion led to prolonged HSPB1 induction, which facilitated astroglial autophagy via two different signaling pathways following KA injection (Fig. 8).

Recently, we have reported that sustained HSPB1 expression triggers astroglial autophagy in the rat PILO-induced SE model^12^. Indeed, HSPB1 activates the autophagy-lysosomal pathway in abnormal astrocytes containing Rosenthal fibers^46^. In the present study, *P2rx7* deletion and P2RX7 antagonists leads to prolonged HSPB1 induction with enhanced LAMP1 expression in astrocytes following KA-induced SE. *P2rx7* deletion also exerts upregulations of ATG5-ATG12, ATG12, and MAP1LC3B expression in astrocytes, which is effectively abrogated by *Hspb1* knockdown. In contrast, BzATP leads to astroglial apoptosis in WT mice accompanied by reduced HSPB1 expression following SE. Since autophagy inhibits astroglial apoptosis^23,28–30^, our findings indicate that prolonged HSPB1 induction by *P2rx7* deletion may lead to astroglial autophagy following KA injection.

*P2rx7* deletion and pharmacological blockade of P2RX7 increase the seizure susceptibility in response to PILO (not KA)^16,47^. The present study also reveals that KO mice showed seizure activity in response to subconvulsive dose of PILO to WT animals, and a lethal dose of PILO to KO mice effectively induced SE in WT mice. Therefore, we could not directly compare the effects of PILO-induced seizure activity on astroglial autophagy and HSPB1 induction between WT and KO mice. However, astroglial HSPB1 and LAMP1 expressions in KO mice were higher than those in WT mice, although the total power of EEG during SE in WT mice was larger than that in KO mice following PILO injection. Furthermore, KA upregulated HSPB1 and LAMP1 expressions in KO mice more than in WT mice without changed seizure susceptibility. In contrast to the present and our previous report^16^, Engel and his colleagues have reported that *P2rx7* deletion or P2RX7 antagonism attenuated the seizure severity induced by intra-amygdala KA injection^48^. If P2RX7 have pro-convulsive effect in response to KA, prolonged HSPB1 induction would be observed in WT mice rather than KO mice in the present study. Regardless of the pro- or anti-convulsive role of P2RX7, our findings indicate *that P2rx7* deletion may result in prolonged HSPB1 induction, independent of seizure activity.

P2RX7 stimulates the release of autophagolysosomes/phagolysosomes from the microglia^49^. Astrocytes also release HSPB1^50^, and impaired clearance of HSPB1 decreases astroglial viability^23^. Thus, the increased HSPB1 protein level induced by *P2rx7* deletion would be interpreted as the consequence from dysfunction of HSPB1 release from astrocytes. In the present study, however, *P2rx7* deletion increased *Hspb1* mRNA and its protein level. Although it does not exclude the possibility that decreased HSPB1 degradation could also contribute to the elevated HSPB1 expression, our findings indicate that *P2rx7* deletion may increase astroglial HSPB1 protein level via enhanced *Hspb1* mRNA transcription.

P2RX7 activates MAPK1/2^44,51,52^, which inhibits HSPB1 expression via SP1-T739 phosphorylation^42,43,53^. In the present study, *P2rx7* deletion reduces MAPK1/2 and SP1 phosphorylations after KA injection. However, *P2rx7* deletion facilitates EIF2AK3/EIF2S1/ATF4 signaling pathway in response to KA, which is attenuated by *Hspb1* siRNA and MMA. Since this signaling pathway replenishes the autophagosome-associated protein pool to allow cells to induce autophagy^54,55^, prolonged HSPB1 induction due to impaired P2RX7-MAPK1/2 pathway may trigger ER stress-mediated astroglial autophagy. Indeed, U0126 results in KA-induced persistent HSPB1 expression and facilitates astroglial autophagy in WT mice. Therefore, our findings suggest that P2RX7-MAPK1/2-SP1 signaling axis may play an important role in the regulation of an optimal astroglial HSPB1 expression.

PRKAA1 is a key player in maintaining energy homeostasis in response to cellular stress. Reduced ATP level activates PRKAA1 by T172 phosphorylation and promotes autophagy via MTOR phosphorylation. In addition, PRKAA1 directly activates autophagy by ULK1 phosphorylation in response to fluctuations in the AMP:ATP ratio^56.57^. In the present study, *P2rx7* deletion activates PRKAA1/ULK1-mediated astroglial autophagy independent of MTOR following KA treatment, which is abrogated by *Hspb1* knockdown. Upregulation of HSPB1 is an indicative of increase in energy consumption^21^, and ER stress-induced EIF2AK3/EIF2S1/ATF4 activation causes ATP depletion and PRKAA1 activation^58,59^. Therefore, the energy-deficient condition by prolonged HSPB1 induction may directly lead to PRKAA1/ULK1-mediated astroglial autophagy, independent of MTOR.

In the present study, KA cannot change the phosphorylations of PIK3, AKT1-T308, AKT1-T450, AKT1S1, MTOR, and RPS6KB1 in both WT and KO mice. Unexpectedly, we found that *P2rx7* deletion-induced PIK3-independent AKT1-S473 phosphorylation after KA treatment, which was abolished by *Hspb1* knockdown. AKT1 is one of the downstream targets of PIK3, which phosphorylates AKT1S1 and relieves AKT1S1-mediated MTOR inhibition^60^. However, PIK3-independent AKT1 activation has been also reported^61–63^. Indeed, HSPB1 increases AKT1-S473 phosphorylation by an unknown mechanism^35,36^. Furthermore, HSP1B forms a complex with AKT1 and maintains its activity^63–66^. Although, it is unclear that HSP1B is an indispensable factor for AKT1 activation in the present study, it is likely that HSP1B may change the conformation of AKT1 for its activation upon stress stimulation.

Since AKT1 phosphorylation is regulated by P2RX7-mediated PTEN activation^67^, *P2rx7* deletion would affect PTEN-mediated AKT1 activity, independent of HSPB1 expression and PIK3 activity. However, we found that *P2rx7* deletion did not affect PTEN expression/phosphorylation following KA injection. Therefore, our findings indicate that *P2rx7* deletion-induced AKT1 activation may be divergent from PIK3- or PTEN-mediated pathway. Analyses of the precise nature of AKT1 activation and its putative kinases will be helpful to understand the mechanism of AKT1-mediated autophagy and the role of HSP1B-AKT1 interaction.

SH3GLB1 is involved in GSK3B inhibitor-induced autophagic response^34^, since SH3GLB1 modulates autophagy by regulating autophagosome formation^68^. Considering GSK3B-S9 phosphorylation by AKT1^69^, it is likely that *P2rx7* deletion-mediated AKT1-S472 phosphorylation may also activate astroglial autophagy after KA injection. Indeed, *P2rx7* deletion reduced GSK3B activity and upregulated SH3GLB1 expression in astrocytes after KA treatment, which were reversed by *Hspb1* knockdown and 3CAI. Therefore, our findings indicate that *P2rx7-*deletion-mediated prolonged HSPB1 induction may also promote AKT1/GSK3B/SH3GLB1 autophagic pathway.

In conclusion, the present study provides novel purinergic signaling pathways that regulate ER stress and autophagy in astrocytes following KA injection (Fig. 8). *P2rx7* deletion impaired MAPK1/2 phosphorylation and increased SP1-mediated HSPB1 transactivation after KA injection, which developed ER stress. ATP depletion induced by ER stress and persistent HSPB1 expression triggered astroglial autophagy via PRKAA1/ULK1 and AKT1/GSK3B/SH3GLB1 cascades. Therefore, our findings suggest that P2RX7 may play an inhibitory role in the regulation of ER stress and autophagy in astrocytes by the fine-tuning of HSPB1 expression under pathophysiological conditions.

## Methods

### Experimental animals and chemicals

We used male C57BL/6J (*P2rx7*^*+/+*^) and *P2rx7*^*−/−*^ mice (60- to 90-day-old, 25–30 g, The Jackson Laboratory, USA) in the present study. Animals were given a commercial diet and water ad libitum under controlled conditions (22 °C ± 2 °C, 55% ± 5% humidity, and 12-h light/12-h dark cycle). Animal protocols were approved by the Institutional Animal Care and Use Committee of Hallym University (Chuncheon, Korea). All reagents were obtained from Sigma-Aldrich, unless otherwise indicated. Supplementary table 1 is a list of the primary antibodies used in the present study.

### Seizure induction and infusions of drug and siRNA oligonucleotide

Mice were given a single dose of KA (25 mg/kg, i.p.) or PILO (200 and 350 mg/kg, i.p.)^16^. To reduce peripheral cholinergic effects, scopolamine methylbromide (2 mg/kg, i.p.) was administered 20 min before PILO. As controls, mice were treated with saline, instead of KA or PILO^16^. Two hours after KA or PILO injection, animals received diazepam (10 mg/kg, i.p.) to terminate seizures. Two days after KA injection, mice were anesthetized with Isoflurane (1–2% in O_2_ and N_2_O) and placed in a stereotaxic frame. Each animal was implanted with a brain infusion kit 3 (Alzet, USA) inserted into the lateral cerebral ventricle 1.0 mm lateral to the bregma and connected to an Alzet osmotic minipump (model 1007D, USA) containing each compound or siRNA; vehicle, BzATP (a P2RX7 agonist, 5 mM), OxATP (a P2RX7 antagonist, 5 mM), A740003 (a P2RX7 antagonist, 10 μM, Santa Cruz Biotechnology Inc.), Comp C (100 nM), 3CAI (25 μM), MMA (25 μM), U0126 (25 μM), mouse *Hspb1* siRNA (Sense, 5′- AAUAAAAGUUGCAAGCUACUU-3′; antisense, 5′-GUAGCUUGCAACUUUUAUUUU-3′); or a nonsilencing (control) RNA. The doses of drugs and siRNA were chosen based on our previous and preliminary studies, indicating that administration of up to the chosen dose was well tolerated, and no signs of neurotoxicity (hind-limb paralysis, vocalization, food intake, or neuroanatomical damage) were observed.

### Electrophysiology

Under Isoflurane anesthesia (1–2% in O_2_ and N_2_O), animals were stereotaxically implanted with a monopolar electrode into the left dorsal hippocampus (−2.0 mm posterior, 1.5 mm lateral, 2.0 mm depth). Three days after surgery, mice were given a single dose of KA or PILO aforementioned after establishing a stable baseline for at least 30 min. EEG signals were acquired using LabChart Pro v7 (AD Instruments, NSW, Australia), and latency or seizure onset and total power were measured from each animal^15^.

### Astroglial isolation

In a preliminary study, we had found that the amount of isolated astrocytes obtained from the bilateral hippocampi of a mouse was not enough for western blot. Thus, we used the bilateral cerebral cortices of an individual mouse to purify the astrocytes. Mice were anesthetized and decapitated. Bilateral cerebral cortices were removed and dissociated with adult brain dissociation kit (Miltenyi biotec, Germany, #130-107-677), according to the manufacturer’s protocol. Thereafter, astrocytes were isolated with anti-astrocyte specific cell surface antigen 2 (ACSA-2) kit (Miltenyi biotec, Germany, #130-097-678), according to the manufacturer’s guidelines. Briefly, up to 10^5^ dissociated cells were suspended in 150 μl 0.5% BSA in PBS buffer and incubated with ACSA-2 microbeads for 25 min at 4°C. Then, the cells were applied to a MS column fitted in a magnetic cell separator. Following column removal from the magnetic separator, astrocytes were eluted in 1.5 ml buffer.

### Western blot

At designated time window, the hippocampus was obtained under urethane anesthesia (1.5 g/kg, i.p.). Tissues and isolated astrocytes were homogenized, and the protein concentration in the supernatant was determined using a Micro BCA Protein Assay Kit (Pierce Chemical, Rockford, IL, USA). Western blot was performed by the standard protocol. Briefly, the aliquots were loaded into a polyacrylamide gel. After electrophoresis, gels were transferred to nitrocellulose transfer membranes. Membranes were incubated with primary antibody. Thereafter, membranes were reacted with a HRP-conjugated secondary antibody and ECL kit (GE Healthcare, Piscataway, NJ, USA). The bands were detected and quantified on ImageQuant LAS4000 system (GE Healthcare, Piscataway, NJ, USA). The rabbit anti-β-actin primary antibody (1:5000) was used as an internal reference. Intensity measurements were represented as the mean gray-scale value and normalized against β-actin.

### Double immunofluorescence study

Control and test animals were perfused via the ascending aorta with 200 ml of 4% paraformaldehyde in phosphate buffer (PB). The brains were removed and cryoprotected by infiltration with 30% sucrose overnight. Thereafter, the tissues were sectioned with a cryostat at 30 μm thickness and the consecutive sections were collected in 6-well plates containing PBS. Sections were incubated in a mixture of antisera (glial fibrillary acidic protein (GFAP) + HSPB1, GFAP + LAMP1, GFAP + p-ULK1-S555, or GFAP + SH3GLB1) in PBS containing 0.3% Triton X-100 overnight at room temperature. After washing, the sections were incubated in a mixture of FITC- and Cy3-conjugated IgG (or streptavidin, Jackson Immunoresearch Laboratories Inc., USA; diluted 1:250) for 2 h at room temperature. In addition, TUNEL staining was performed with the TUNEL apoptosis detection kit (Upstate, Lake Placid, NY, USA), according to the manufacturer’s protocol (http://www.upstate.com). Following the TUNEL reaction, double fluorescent staining was performed as the same methods aforementioned. To establish the specificity of immunostaining, a negative control test was carried out with pre-immune serum, instead of the primary antibody. No immunoreactivity was observed for the negative control in any structures. All experimental procedures in this study were performed under the same condition and in parallel. Images were captured using an AxioImage M2 microscope. Fluorescent intensity was measured using computer-based image analysis program (AxioVision Rel. 4.8 software, Germany). Fluorescent intensity was then standardized by setting the threshold level (mean background intensity obtained from 5 image input). Manipulation of the images was restricted to threshold and brightness adjustments to the whole image. In addition, images of the hippocampus were captured (five sections per each animal), and the areas of interest (1 × 10^5^ μm^2^) were selected. Thereafter, two different investigators performed TUNEL-positive cell counts.

### Statistics

All data were analyzed using two-tailed Student’s *t*-test or one-way ANOVA to determine statistical significance. Bonferroni’s test was used for post-hoc comparisons. A *p*-value <0.05 was considered statistically significant^15^.

## Electronic supplementary material

Supplementary information
