# Supplementary material for: P2RX7-MAPK1/2-SP1 axis inhibits MTOR independent HSPB1-mediated astroglial autophagy
Source: Cell Death Dis. 2018 May 10;9(5):546. doi: 10.1038/s41419-018-0586-x (PMC5945848; doi:10.1038/s41419-018-0586-x)
Supplement: Supplementary file 1 — Supplementary information [file 41419_2018_586_MOESM1_ESM.pdf]

Supplementary information

P2RX7-MAPK1/2-SP1 axis inhibits MTOR independent HSPB1-mediated astroglial autophagy

Ji-Eun Kim,[1] Ah-Reum Ko,[1] Hye-Won Hyun,[1] Su-Ji Min,[1] and Tae-Cheon Kang[1,*]

[1]*Department of Anatomy and Neurobiology, Institute of Epilepsy Research, College of Medicine, Hallym University, Chunchon 200-702, South Korea.*

Running title: Roles of P2RX7-mediated HSPB1 induction in autophagy

* Correspondence to: T. -C. Kang, Department of Anatomy and Neurobiology, College of Medicine, Hallym University, Chunchon, Kangwon-Do 200-702, South Korea; Tel: +82-33-248-2524; Fax: +82-33-248-2525; E-mail: tckang@hallym.ac.kr

Grant sponsor: National Research Foundation of Korea (No. 2013R1A6A3A04058272 and 2015R1A2A2A01003539).

Supplementary Table 1. Primary antibodies used in the present study

Antibody	Host	Manufacturer (catalog number)	Dilution used
AKT1	Rabbit	Cell signaling (#9272)	1:1,000 (WB)
AKT1S1	Rabbit	Abcam (ab181408)	1:1,000 (WB)
ATF4	Rabbit	Proteintech (10835-1-AP)	1:1,000 (WB)<br>1:100 (IF)
ATF6	Rabbit	Proteintech (24169-1-AP)	1:1,000 (WB)
ATG12	Rabbit	Genetex (GTX124181)	1:1,000 (WB)
CLDN11 (Claudin 11)	Mouse	Millipore (MAB1580)	1:5,000 (IF)
DDIT3	Mouse	Cell signalling (#2895)	1:1,000 (WB)
GFAP	Mouse	Millipore (mab3402)	1:1,000 (WB)<br>1:4,000 (IF)
GSK3B	Rabbit	Elapscience (ENT2082)	1:1,000 (WB)
HSF1	Rabbit	Proteintech (51034-1-AP)	1:1,000 (WB)
HSPB1	Rabbit	Enzo (ADI-SPA-801)	1:1,000 (WB)<br>1:500 (IF)<br>1:50 (IP)
ITGAM (Integrin alpha M)	Rat	Bio-Rad (MCA711)	1:100 (IF)
LAMP1	Rabbit	Lifespan (LS-B580)	1:1,000 (WB)<br>1:200 (IF)
MAP1LC3B	Rabbit	Abcam (ab48394)	1:1,000 (WB)
MAPK1/2	Rabbit	Biorbyt (orb160960)	1:1,000 (WB)
MTOR	Rabbit	Cell signalling (2972S)	1:1,000 (WB)
p-PRKAA1-T172	Rabbit	Abcam (ab195946)	1:1,000 (WB)
p-AKT1-S473	Rabbit	Cell signalling (#4060)	1:1,000 (WB)
p-AKT1-T308	Rabbit	Cell signalling (#9275)	1:1,000 (WB)
p-AKT1-T450	Rabbit	Cell signalling (9267S)	1:1,000 (WB)
p-AKT1S1-T246	Rabbit	Abcam (ab134084)	1:1,000 (WB)
p-EIF2AK3-T980	Rabbit	Biorbyt (orb6693)	1:500 (WB)
p-EIF2S1-S51	Rabbit	Sigma (SAB4504388)	1:1,000 (WB)

p-ERN1	Rabbit	Thermo (PA1-16927)	1:1,000 (WB)
p-GSK3B-S9	Rabbit	Biorbyt (orb14745)	1:1,000 (WB)
p-HSF1-S303	Rabbit	Bioworld (BS4795)	1:1,000 (WB)
p-MAPK1/2-Thr202/Tyr204, Thr185/Tyr187	Rabbit	Millipore (#05-797RSP)	1:1,000 (WB)
p-MTOR-S2448	Rabbit	Cell signalling (2971S)	1:1,000 (WB)
p-MTOR-S2481	Rabbit	Cell signalling (2974S)	1:1,000 (WB)
p-PIK3-Y458	Rabbit	Cell signalling (4228S)	1:1,000 (WB)
p-PTEN-S820/T382/383	Rabbit	Cell signalling (#9549)	1:1,000 (WB)
p-RPS6KB1-T389	Rabbit	Cell signalling (#9205)	1:1,000 (WB)
p-SP1-T739	Rabbit	Abcam (ab195733)	1:1,000 (WB)
p-ULK1-S555	Rabbit	Cell signalling (#5869)	1:1,000 (WB) 1:50 (IF)
p-ULK1-S757	Rabbit	Cell signalling (#14202)	1:1,000 (WB)
PIK3	Rabbit	Cell signalling (4292S)	1:1,000 (WB)
PRKAA1	Rabbit	Abcam (ab3759)	1:1,000 (WB)
PTEN	Rabbit	Abcam (ab32199)	1:10,000 (WB)
RBFOX3 (RNA binding protein, fox-1 homolog 3)	Guinea pig	Millipore (#ABN90P)	1:1,000 (IF)
RPS6KB1	Rabbit	Proteintech (14485-1-AP)	1:1,000 (WB)
SH3GLB1	Rabbit	Cell signalling (#4427)	1:1,000 (WB) 1:100 (IF)
SP1	Rabbit	Elapscience (ENT3472)	1:1,000 (WB)
ULK1	Rabbit	Cell signalling (#8054)	1:1,000 (WB)
β-actin	Mouse	Sigma (A5316)	1:5,000 (WB)

IF, Immunofluorescence; WB, Western blot.

Supplementary Figures

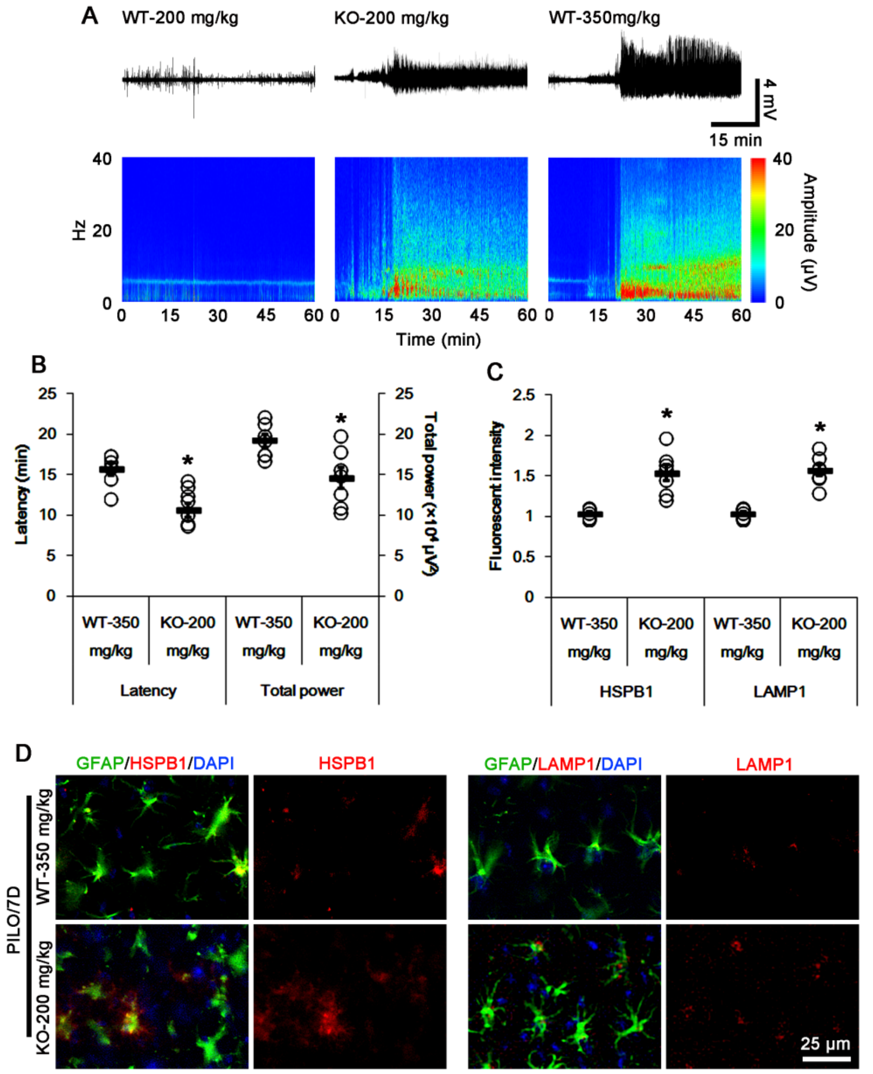

Supplementary Figure 1. Effect of *P2rx7* deletion on HSPB1-mediated autophagy in response to PILO. (A) Representative EEG traces and frequency-power spectral temporal maps in response to PILO. KO mice show the seizure activity in response to subconvulsive dose of PILO (200 mg/kg) to WT animals. (B) Quantification of SE induction, latency and total EEG power in response to PILO. Open circles indicate each individual value. Horizontal bars indicate mean value. Error bars indicate SEM (**p* < 0.05 vs. WT-350 mg/kg; n = 7, respectively). (C) Quantifications of HSPB1 and LAMP1 expression 7 days after PILO injection. Open circles indicate each individual value. Horizontal bars indicate mean value. Error bars indicate SEM (**p* < 0.05 vs. WT-350 mg/kg; n = 7, respectively). (D) Representative photos demonstrating HSPB1 (left panels) and LAMP1 (right panels). Cont, control animals; PILO/7D, 7 days post-PILO injected animals. PILO injection up-regulates HSPB1 and LAMP1 expressions only in the KO mice.

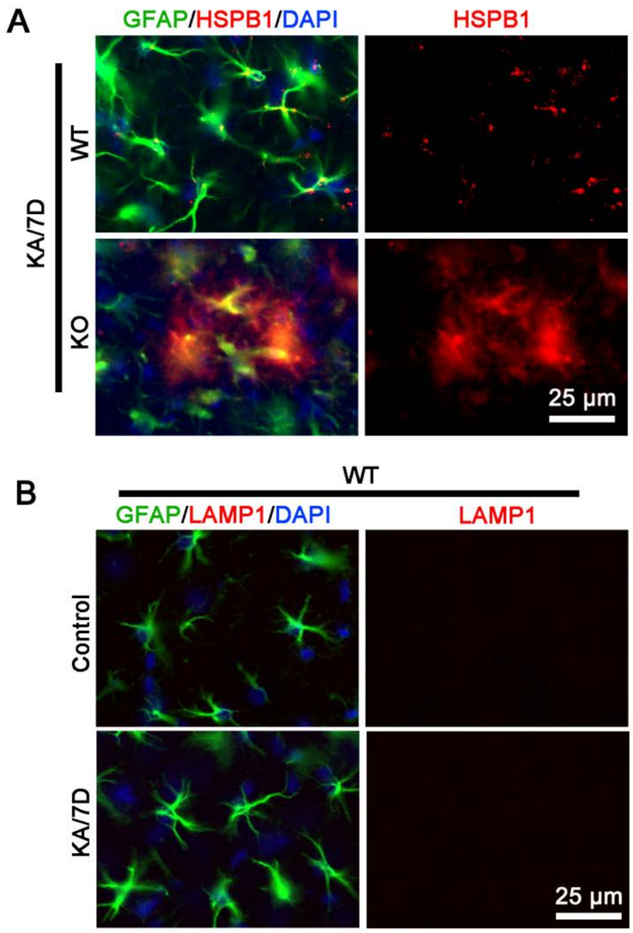

Supplementary Figure 2. Representative photos demonstrating HSPB1 (A) and LAMP1 (B) expression in astrocytes in WT mice 7 days after KA injection (KA/7D). Control, Control animal.

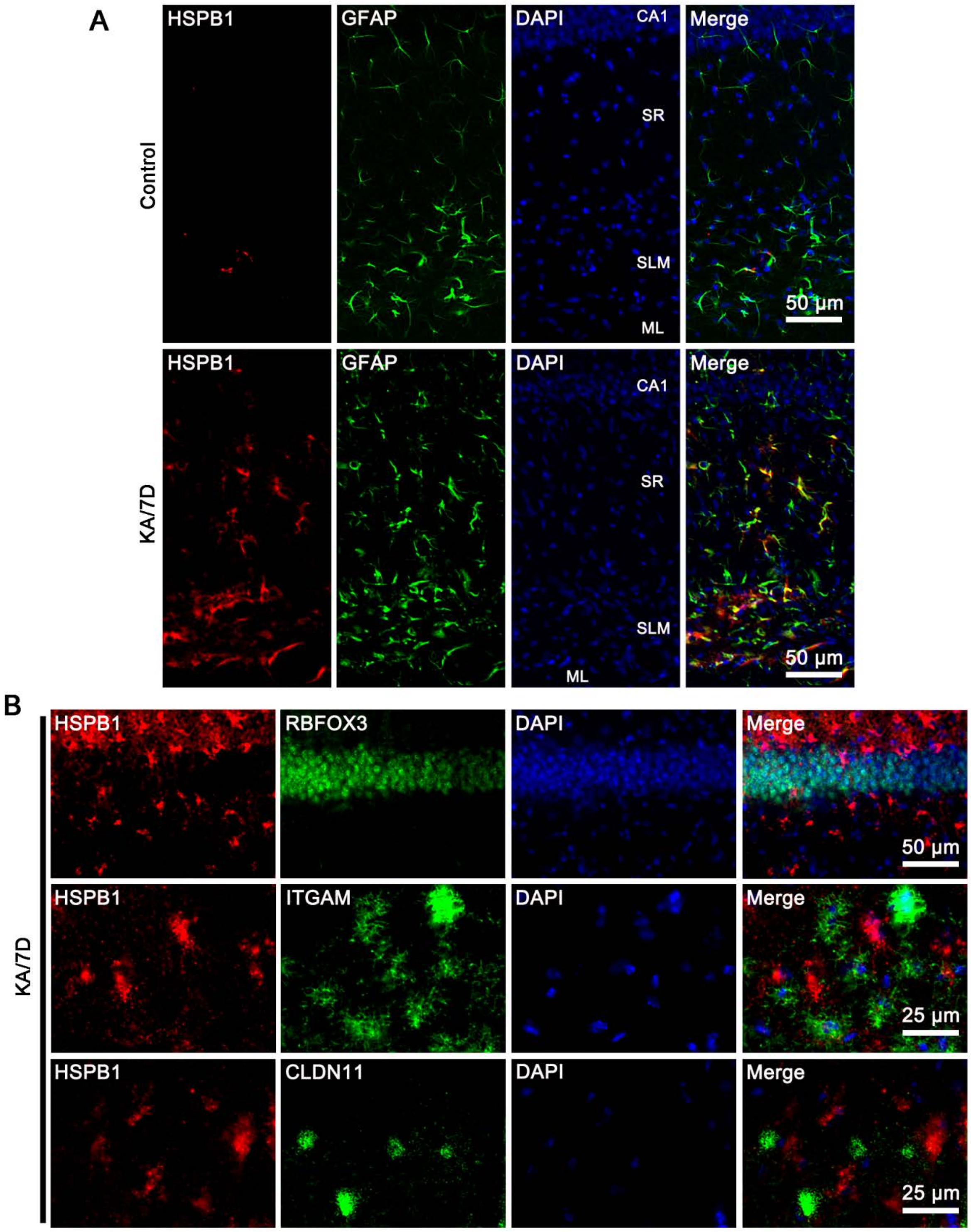

Supplementary Figure 3. Identification of cell population showing HSPB1 induction in the KO mouse hippocampus 7 days after KA injection. *P2rx7* deletion increases HSPB1 expression in GFAP-positive astrocytes (A), not in RBFOX3 (a neuronal marker), ITGAM (a microglial marker) or CLDN11 (an oligodendroglial marker)-positive cells (B).

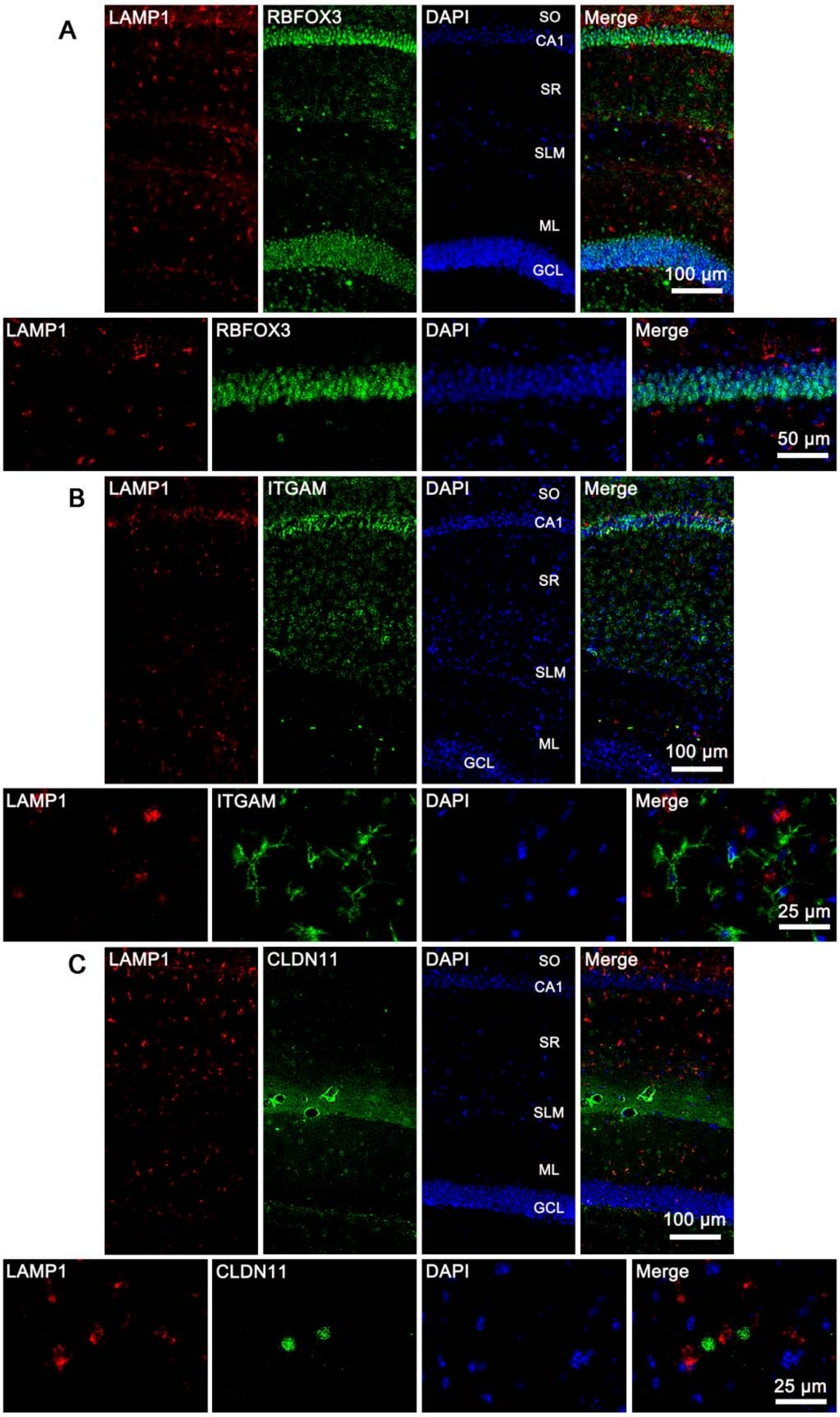

Supplementary Figure 4. Identification of cell population showing up-regulated LAMP1 expression in the KO mouse hippocampus 7 days after KA injection. *P2rx7* deletion cannot increases LAMP1 expression in RBFOX3 (a neuronal marker, A), ITGAM (a microglial marker, B) or CLDN11 (an oligodendroglial marker, C)-positive cells.

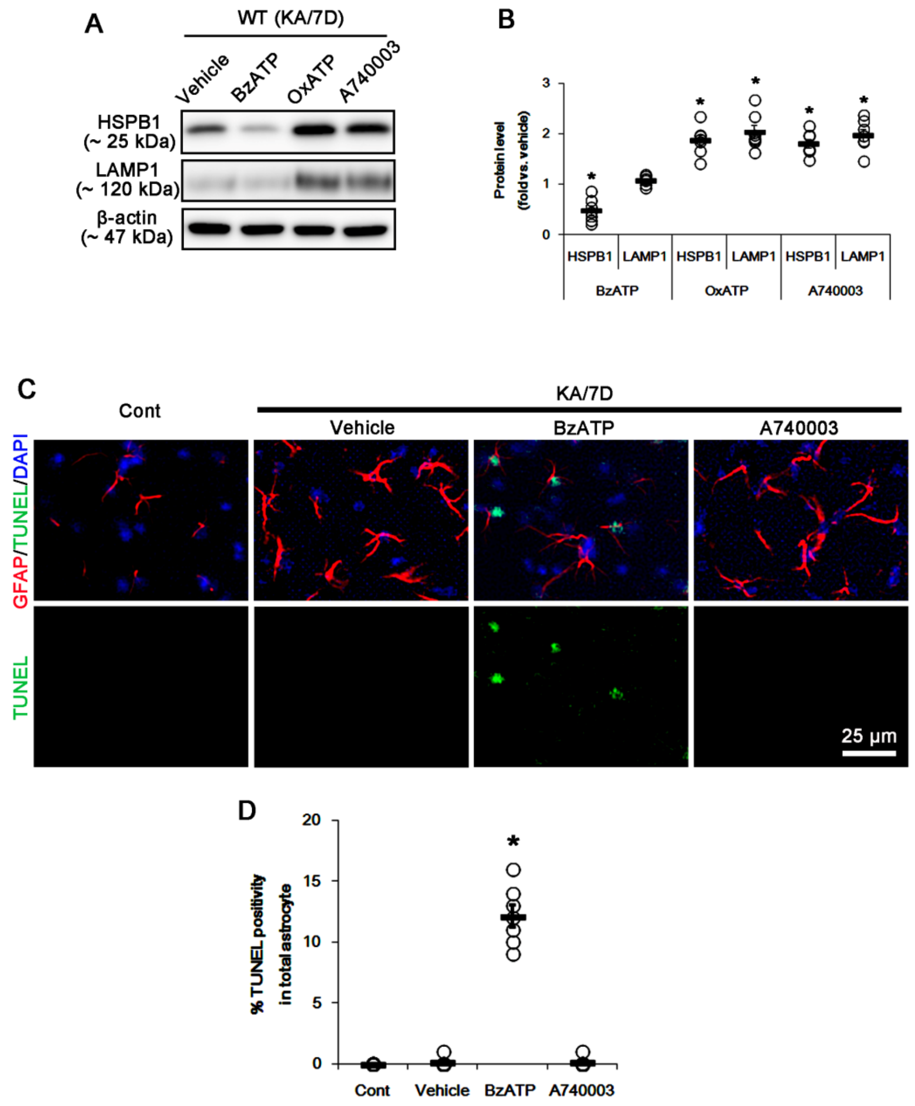

Supplementary Figure 5. (A, B) Effects of P2RX7 agonist and antagonists on HSPB1 and LAMP1 expressions in WT mice 7 days after KA injection (KA/7D). As compared to vehicle, BzATP reduces HSPB1 expression, but not LAMP1 expression. However, both P2RX7 antagonists (OxATP and A740003) significantly elevate HSPB1 and LAMP1 expression levels in WT mice 7 days after KA injection. **(A)** Representative western blot of HSPB1 and LAMP1 in the whole WT mouse hippocampus. **(B)** Quantifications of effect of P2RX7 agonist and antagonists on HSPB1 and LAMP1 protein expressions in WT mice 7 days after KA injection. Open circles indicate each individual value. Horizontal bars indicate mean value. Error bars indicate SEM (*$p < 0.05$ vs. vehicle, respectively; n = 7, respectively). **(C, D)** Effects of P2RX7 agonist and antagonist on astroglial apoptosis in WT mice 7 days after KA injection (KA/7D). **(C)** Representative photos demonstrating TUNEL-positive astrocytes in the WT mice. As compared to vehicle, only BzATP induces astroglial apoptosis following KA injection. **(D)** Quantifications of effect of P2RX7 agonist and antagonists on astroglial apoptosis in WT mice 7 days after KA injection. Open circles indicate each individual value. Horizontal bars indicate mean value. Error bars indicate SEM (*$p < 0.05$ vs. vehicle, respectively; n = 7, respectively).

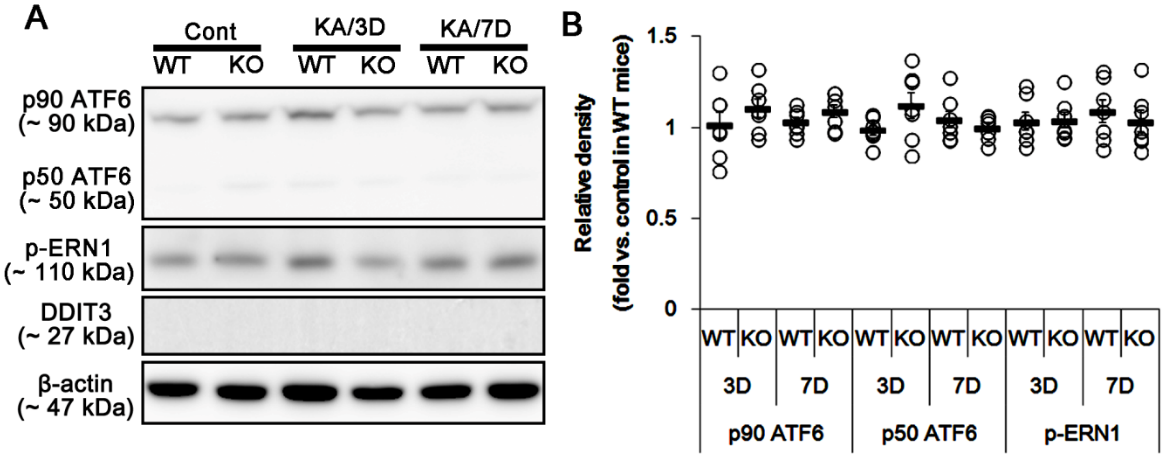

Supplementary Figure 6. Effect of *P2rx7* deletion on DDIT3 and ATF6 expressions and ERN1 phosphorylation following KA injection. There is no difference in p-ERN1, DDIT3 and ATF6 levels between WT and KO mice. (A) Representative western blot of DDIT3 and ATF6 expressions and ERN1 phosphorylation in the whole hippocampus. Cont, control animals; KA/3D, 3 days post-KA injected animals; KA/7D, 7 days post-KA injected animals (B) Quantifications of DDIT3 and ATF6 expressions and ERN1 phosphorylation. Open circles indicate each individual value. Horizontal bars indicate mean value. Error bars indicate SEM (*$p < 0.05$ vs. vehicle, respectively; n = 7, respectively).

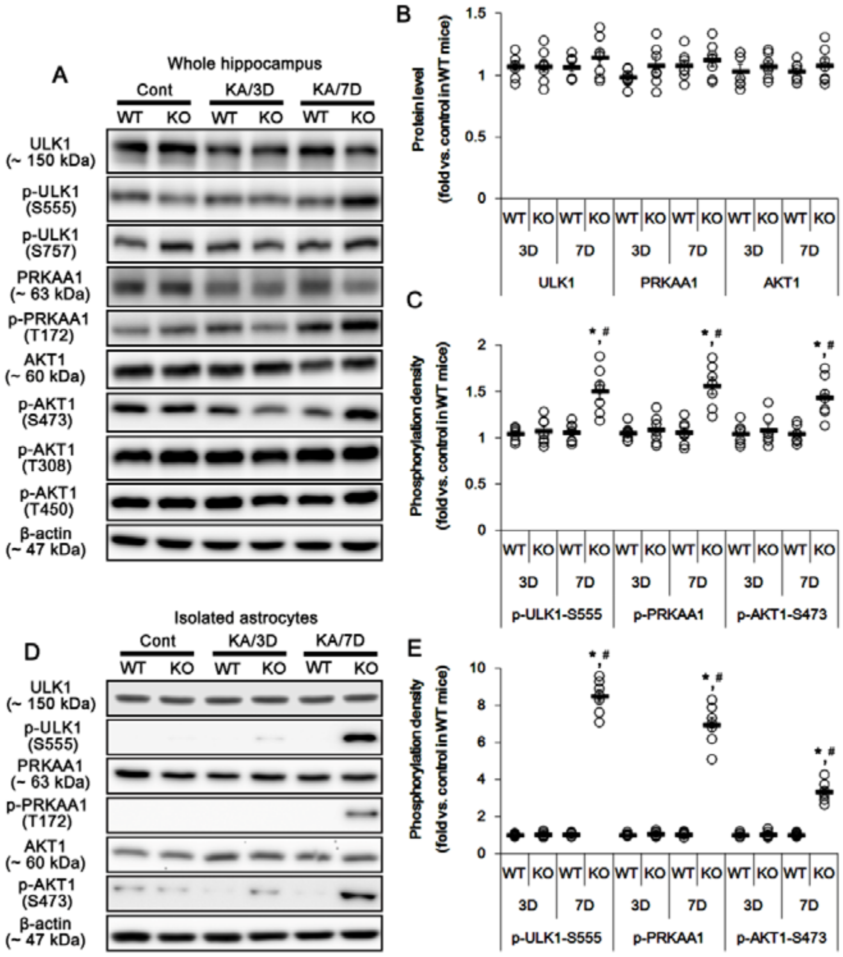

Supplementary Figure 7. PRKAA1/ULK1-mediated astroglial autophagy following KA injection. **(A)** Representative western blot of ULK1-related molecules in the whole hippocampus. As compared to WT mice, KA injection increases ULK1-S555, PRKAA1-T173 and AKT1-S473 phosphorylation in KO mice without altered their expressions. Cont, control animals; KA/3D, 3 days post-KA injected animals; KA/7D, 7 days post-KA injected animals. **(B-C)** Quantifications of expressions (B) and phosphorylations (C) of ULK1-related molecules in the whole hippocampus. Open circles indicate each individual value. Horizontal bars indicate mean value. Error bars indicate SEM ($*,^{\#}p < 0.05$ vs. control and WT mice, respectively; n = 7, respectively). **(D)** Representative western blot of ULK1-related molecules in isolated astrocytes. As compared to WT astrocytes, KA injection increases ULK1-S555, PRKAA1-T173 and AKT1-S473 phosphorylation in *P2rx7*-deleted astrocytes without altered their expressions. **(E)** Quantifications of phosphorylations of ULK1-related molecules in isolated astrocytes. Open circles indicate each individual value. Horizontal bars indicate mean value. Error bars indicate SEM ($*,^{\#}p < 0.05$ vs. control and WT mice, respectively; n = 7, respectively).

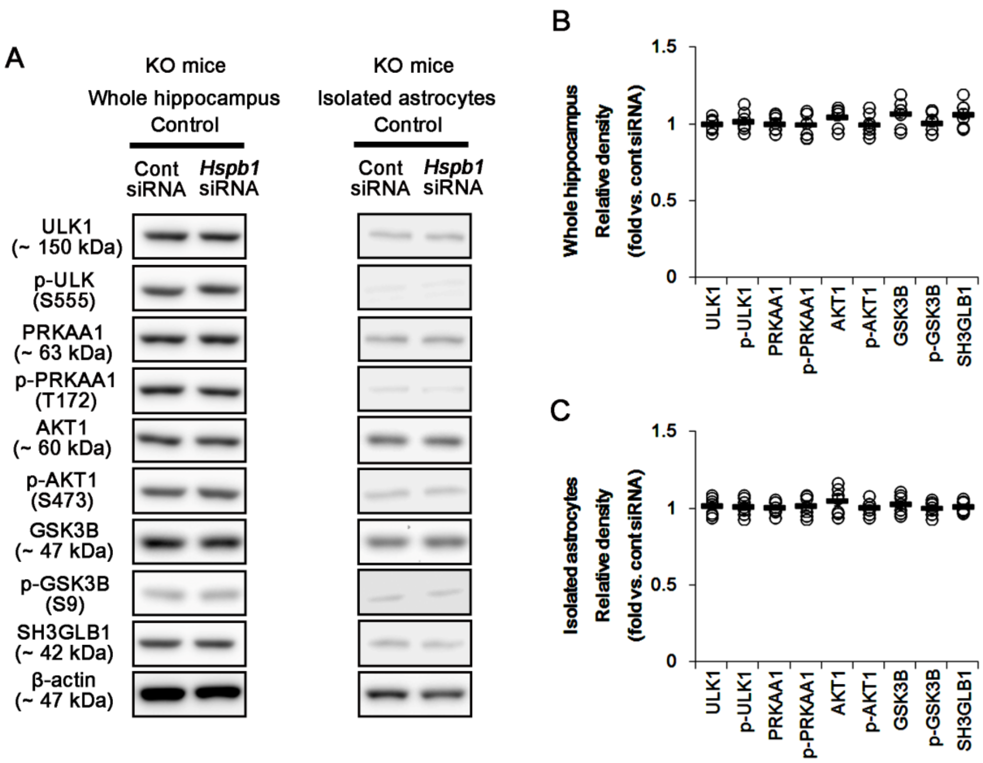

Supplementary Figure 8. Effect of *Hspb1* knockdown on autophagy-related molecules under basal condition (control animals). **(A)** Representative western blot of autophagy-related molecules in the whole KO hippocampus (Left panels) and *P2rx7*-deleted astrocytes (Right panels). *Hspb1* knockdown cannot affect expressions and phosphorylations of autophagy-related molecules. **(B-C)** Quantifications of expressions and phosphorylations of autophagy-related molecules in the whole hippocampus (B) and isolated astrocytes (C). Open circles indicate each individual value. Horizontal bars indicate mean value. Error bars indicate SEM (n = 7, respectively).

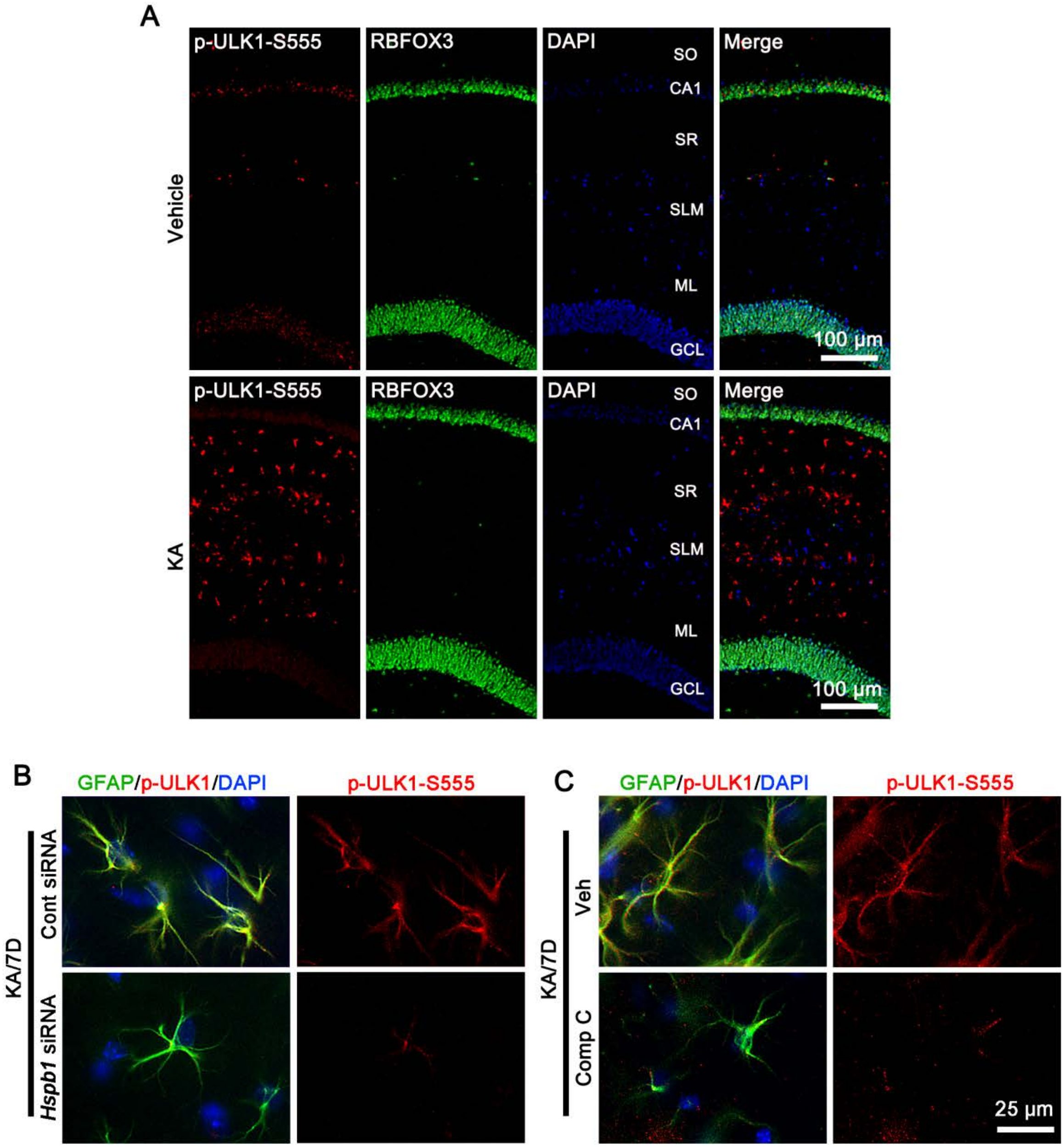

Supplementary Figure 9. Evaluation of neuronal ULK1 phosphorylation (A) and the effects of *Hsp1* knockdown and Comp C on ULK1 phosphorylation (B, C). **(A)** Representative photos demonstrating ULK1-S555 phosphorylation in KO mice. KA cannot increase ULK1 phosphorylation in RBFOX3-positive neurons. **(B, C)** Representative photos demonstrating astroglial ULK1-S555 phosphorylation in KO mice 7 days after KA injection. Both *Hspb1* siRNA (B) and Comp C (C) mitigate ULK1-S555 phosphorylation in astrocytes.

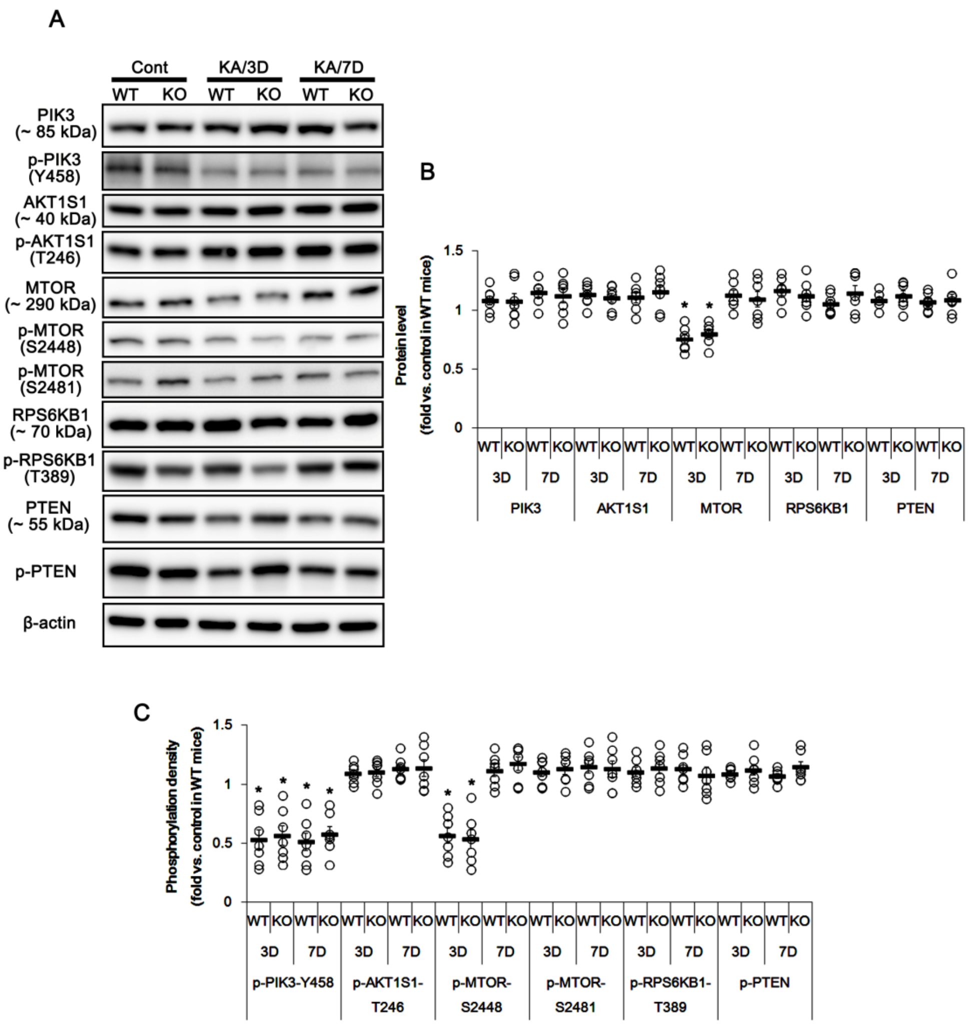

Supplementary Figure 10. Effect of *P2rx7* deletion on PIK3, AKT1S1, MTOR, RPS6KB1 and PTEN expressions and their phosphorylations in the whole hippocampus. PIK3-Y458 phosphorylation is reduced in both WT and KO mice following KA treatment. In addition, MTOR expression and its phosphorylation are reduced in both WT and KO mice 3 days after KA injection. However, there is no difference in PIK3, AKT1S1, MTOR and RPS6KB1 expressions and their phosphorylations between WT and KO mice. **(A)** Representative western blot of PIK3, AKT1S1, MTOR, RPS6KB1 and PTEN expressions and their phosphorylations. Cont, control animals; KA/3D, 3 days post-KA injected animals; KA/7D, 7 days post-KA injected animals. **(B)** Quantifications of PIK3, AKT1S1, MTOR, RPS6KB1 and PTEN expressions. Open circles indicate each individual value. Horizontal bars indicate mean value. Error bars indicate SEM (*$p < 0.05$ vs. control; n = 7, respectively). **(C)** Quantifications of PIK3, AKT1S1, MTOR, RPS6KB1 and PTEN phosphorylations (*$p < 0.05$ vs. control; n = 7, respectively).

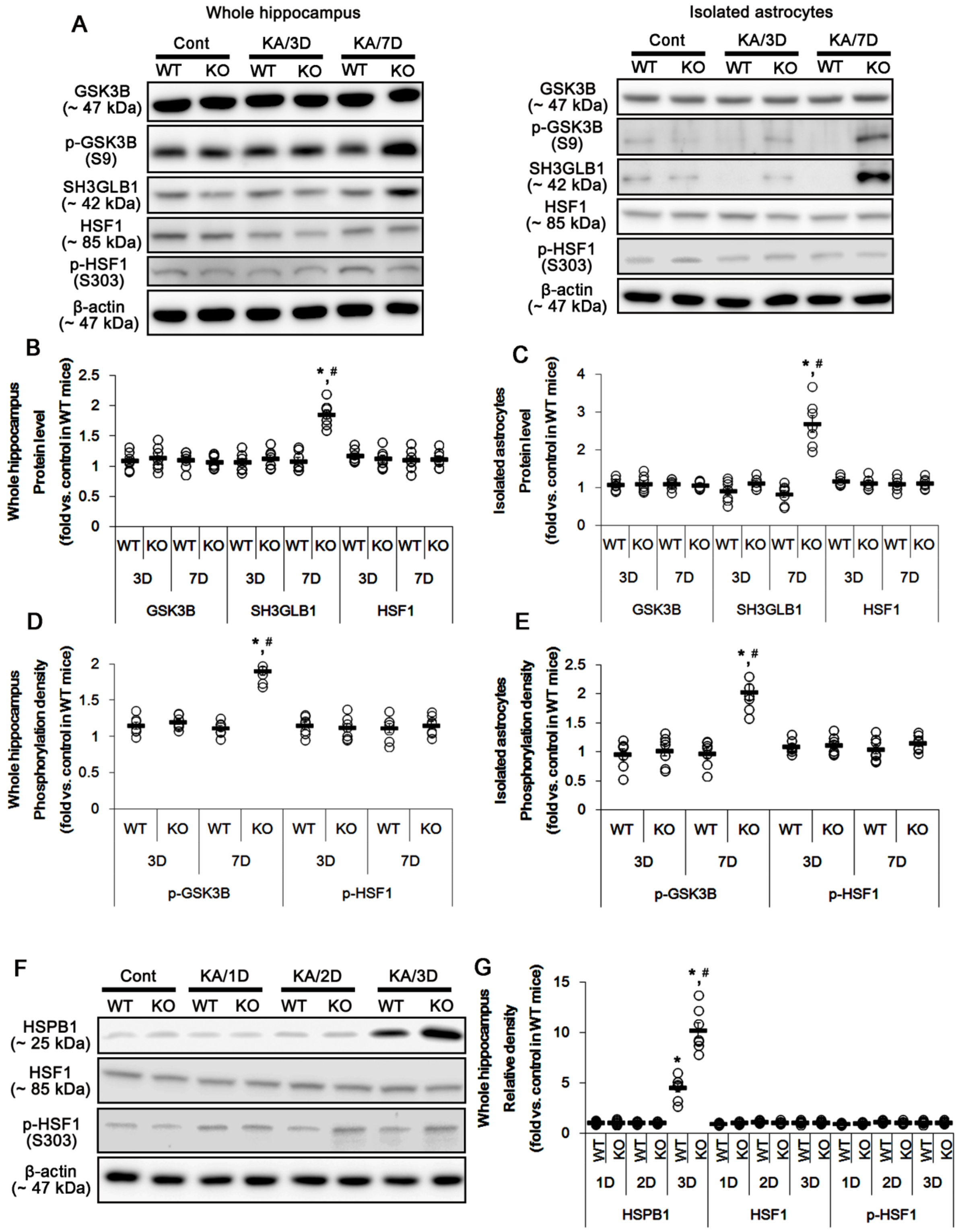

Supplementary Figure 11. AKT1-GSK3B/SH3GLB1-mediated autophagy following KA injection. **(A)** Representative western blot of GSK3B, SH3GLB1 and HSF1 expression and their phosphorylations in the

whole hippocampus (Left panels) and isolated astrocytes (Right panels). KA injection increases GSK3B-S9 phosphorylation and SH3GLB1 expression in KO mice without altered HSP1 expression and its phosphorylation. Cont, control animals; KA/3D, 3 days post-KA injected animals; KA/7D, 7 days post-KA injected animals. **(B-C)** Quantifications of GSK3B, SH3GLB1 and HSF1 expressions in the whole hippocampus (B) and isolated astrocytes (C). Open circles indicate each individual value. Horizontal bars indicate mean value. Error bars indicate SEM ($*,^{\#}p < 0.05$ vs. control and WT mice, respectively; n = 7, respectively). **(D-E)** Quantifications of GSK3B and HSF1 phosphorylations in the whole hippocampus (D) and isolated astrocytes (E). Open circles indicate each individual value. Horizontal bars indicate mean value. Error bars indicate SEM ($*,^{\#}p < 0.05$ vs. control and WT mice, respectively; n = 7, respectively). **(F)** Representative western blot of HSPB1 expressions and HSF1 expression/phosphorylation in the whole hippocampus at the early time points (1 and 2 day-post KA injection). There is no difference in HSF1 expresssion/phosphorylation among groups. **(G)** Quantifications of HSPB1 expressions and HSF1 expression/phosphorylation in the whole hippocampus. Open circles indicate each individual value. Horizontal bars indicate mean value. Error bars indicate SEM ($*,^{\#}p < 0.05$ vs. control and WT mice, respectively; n = 7, respectively).

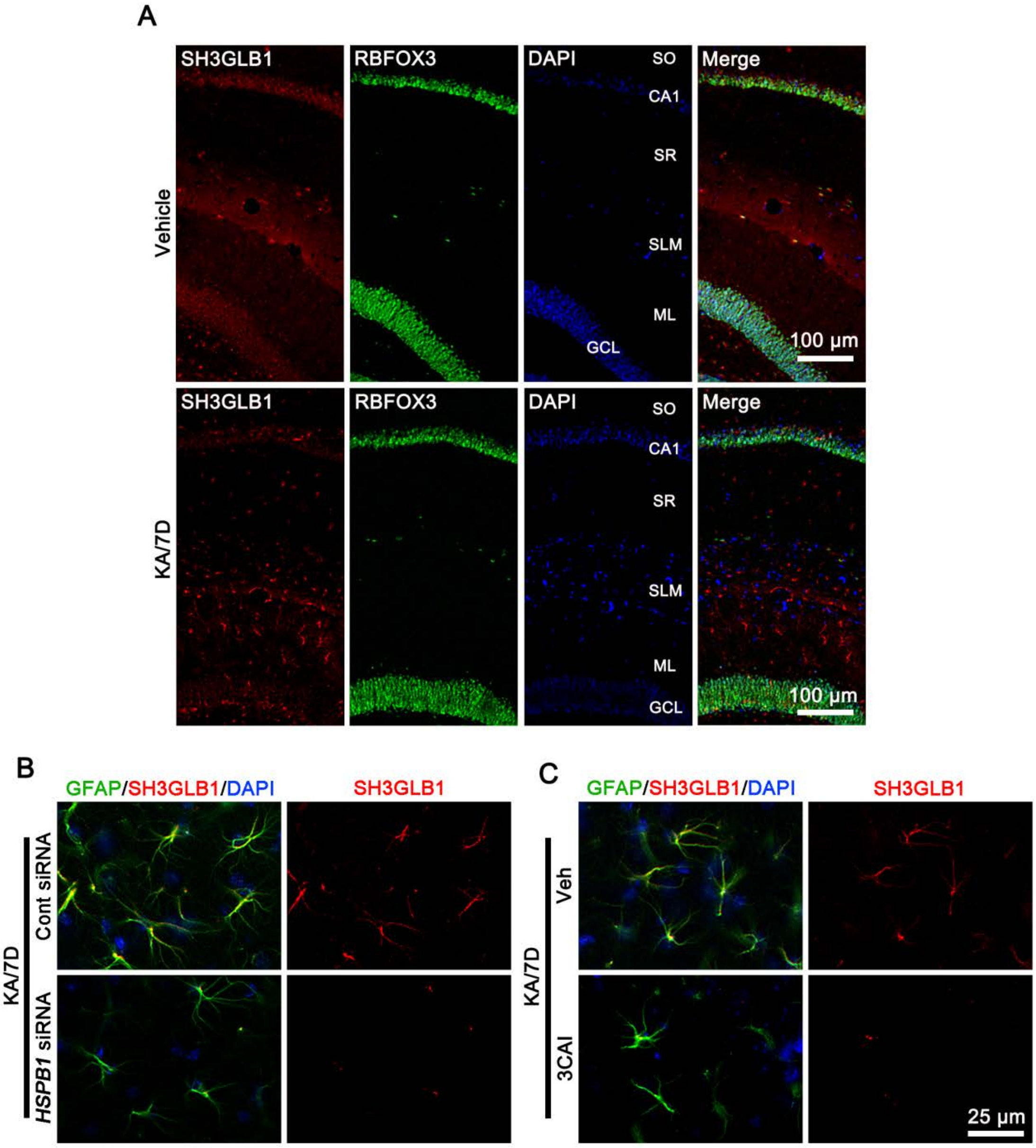

Supplementary Figure 12. Evaluation of neuronal SH3GLB1 expression (A) and the effects of *Hsp1* knockdown and 3CAI on SH3GLB1 expression (B, C). **(A)** Representative photos demonstrating SH3GLB1 expression in KO mice. KA cannot alter SH3GLB1 expression in RBFOX3-positive neurons. **(B, C)** Representative photos demonstrating astroglial SH3GLB1 expression in KO mice 7 days after KA injection. Both *Hspb1* siRNA (B) and Comp C (C) attenuate SH3GLB1 expression in astrocytes.

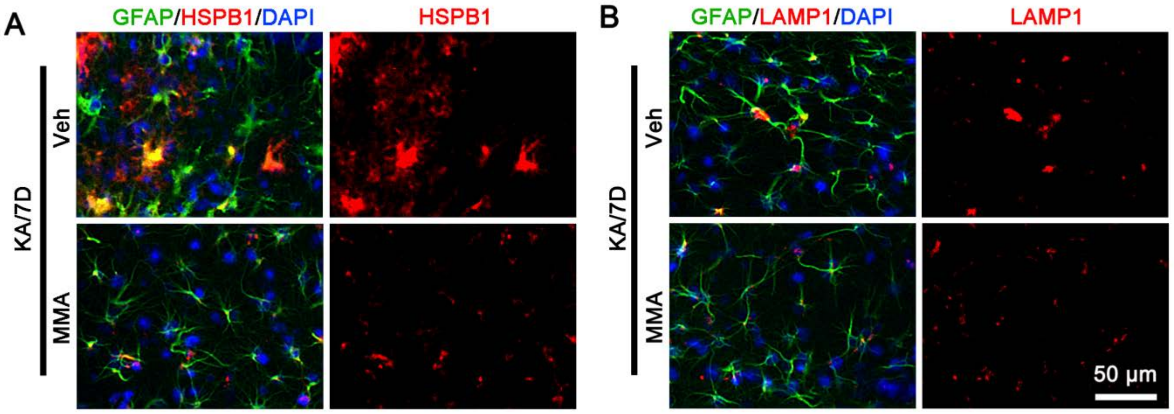

Supplementary Figure 13. Representative photos demonstrating the effect of MMA on HSPB1 (A) and LAMP1 (B) expression in KO mice 7 days after KA injection. MMA abolishes both HSPB1 and LAMP1 expression in astrocytes. Bar = 25 μm.

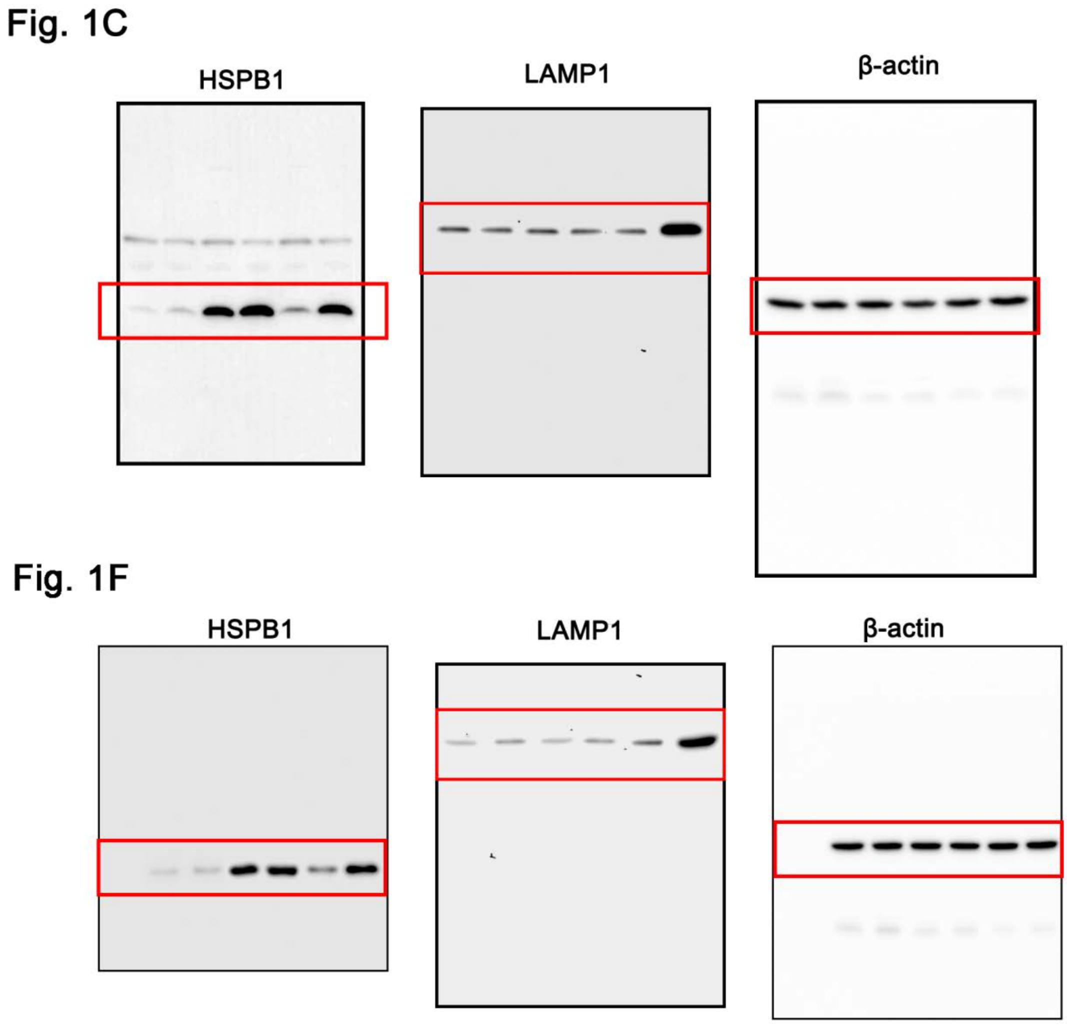

Supplementary Figure 14. Full-length gel images of western blot data in Fig. 1. The cropped parts of western blots are indicated with boxes.

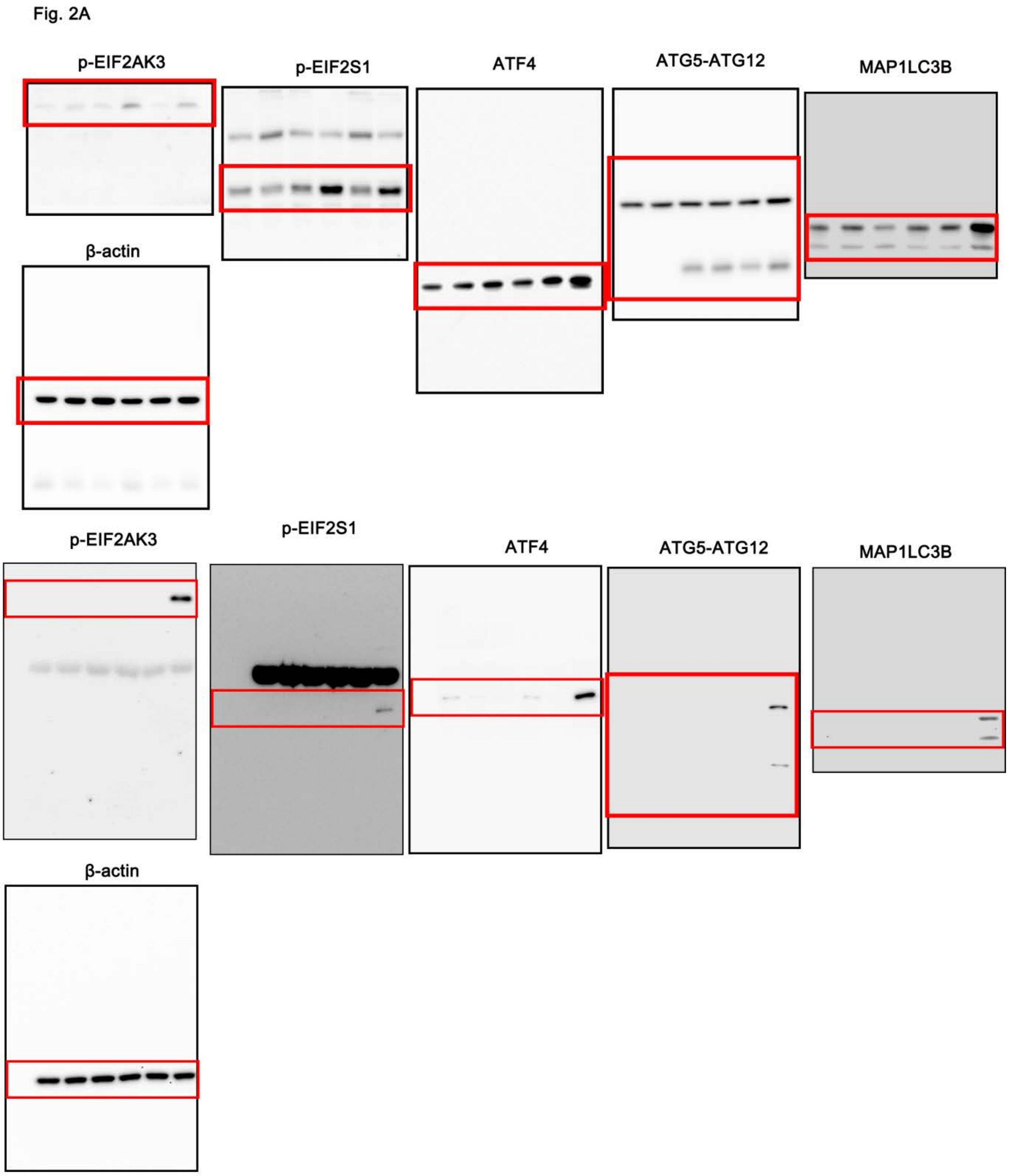

Supplementary Figure 15. Full-length gel images of western blot data in Fig. 2. The cropped parts of western blots are indicated with boxes.

Fig. 3A

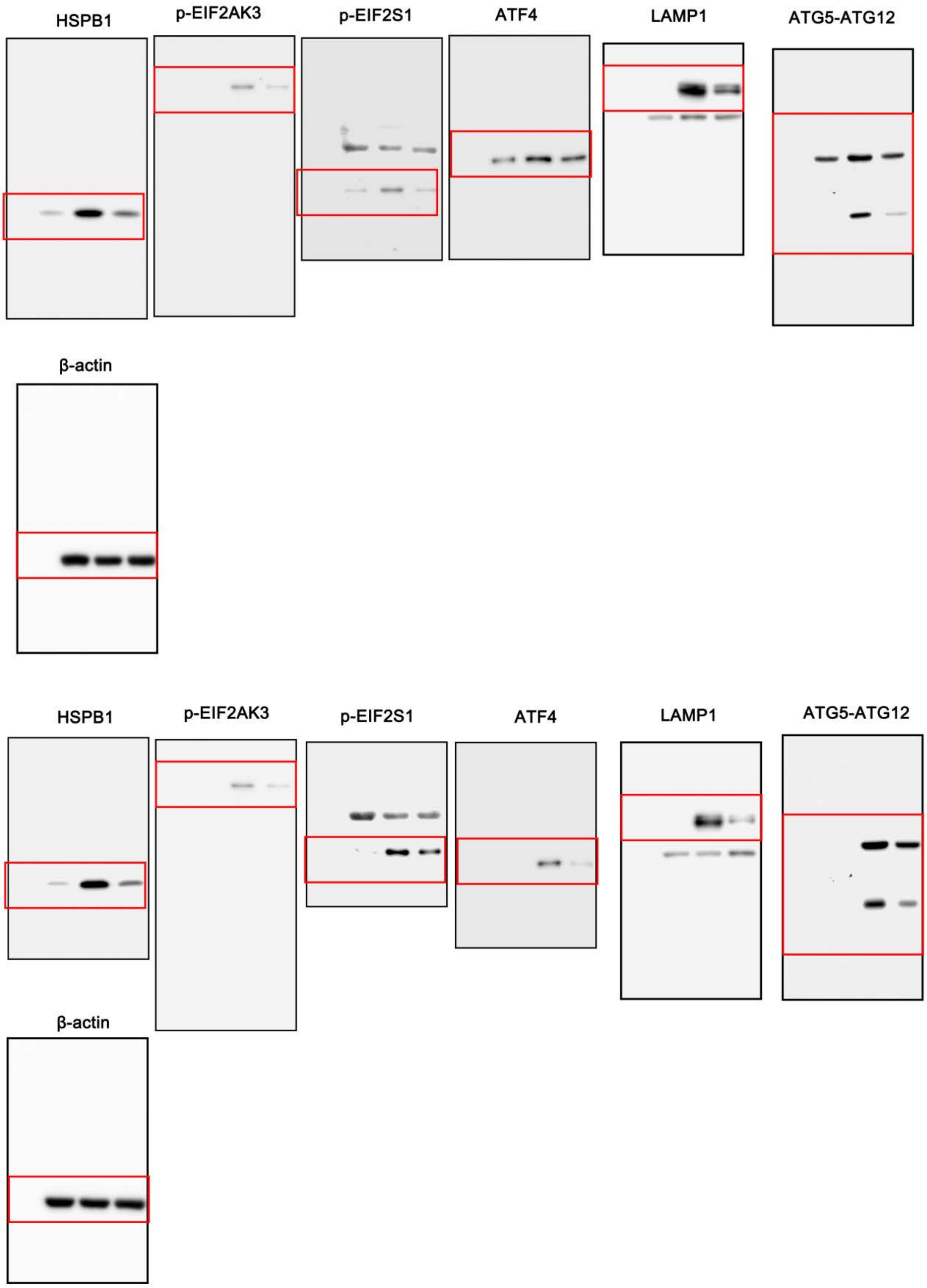

Supplementary Figure 16. Full-length gel images of western blot data in Fig. 3. The cropped parts of western blots are indicated with boxes.

Fig-4

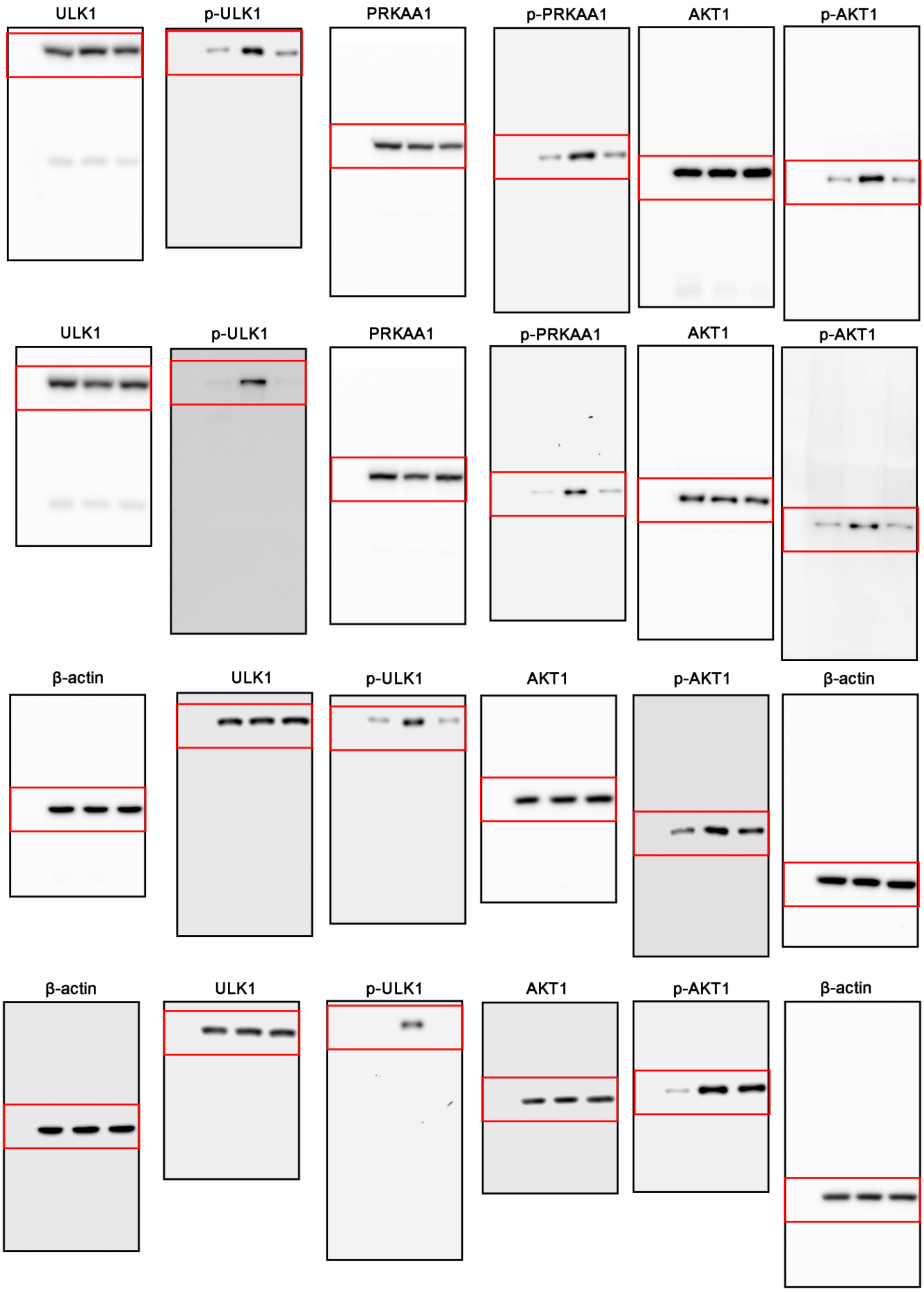

Supplementary Figure 17. Full-length gel images of western blot data in Fig. 4. The cropped parts of western blots are indicated with boxes.

Fig-5

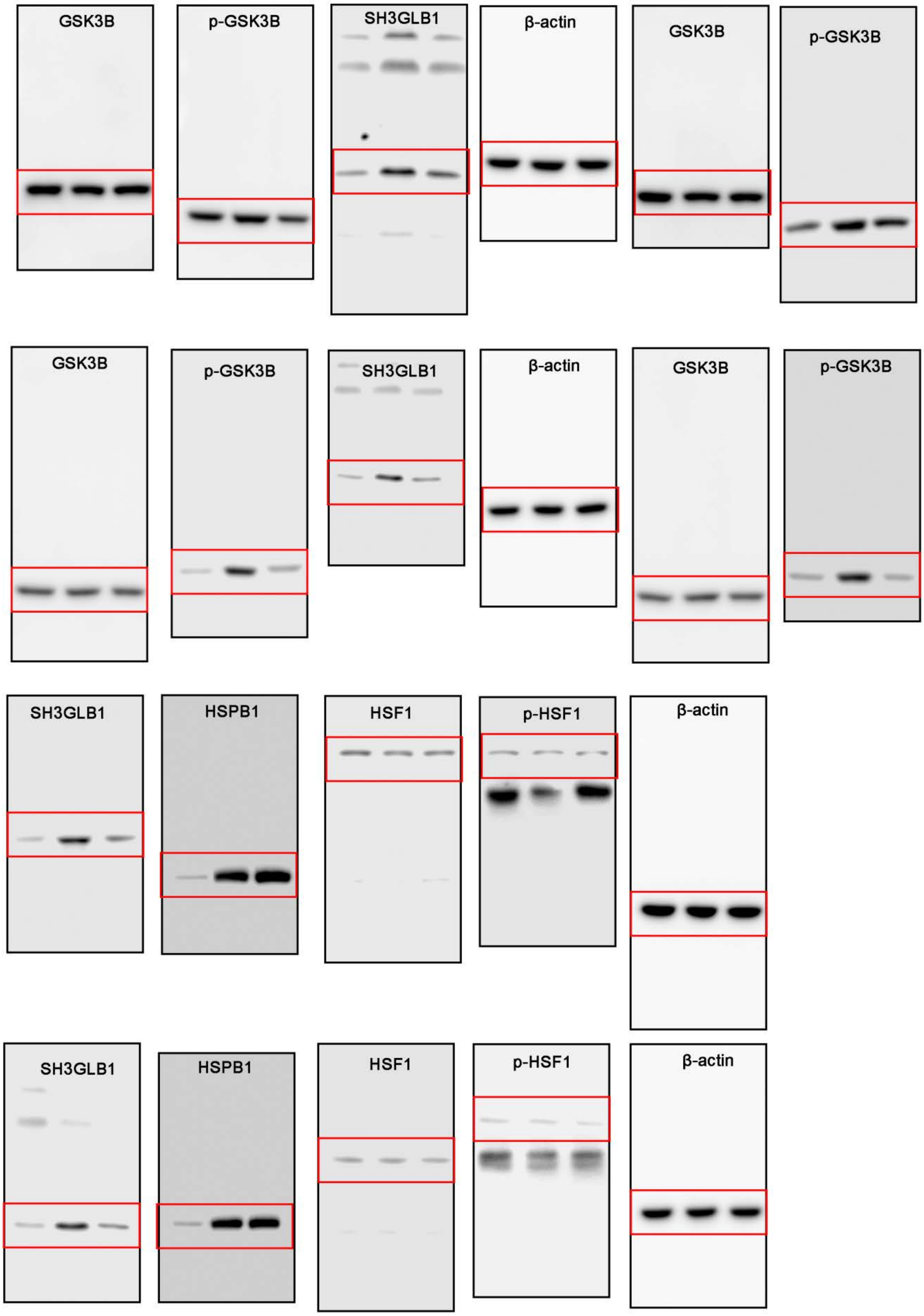

Supplementary Figure 18. Full-length gel images of western blot data in Fig. 5. The cropped parts of western blots are indicated with boxes.

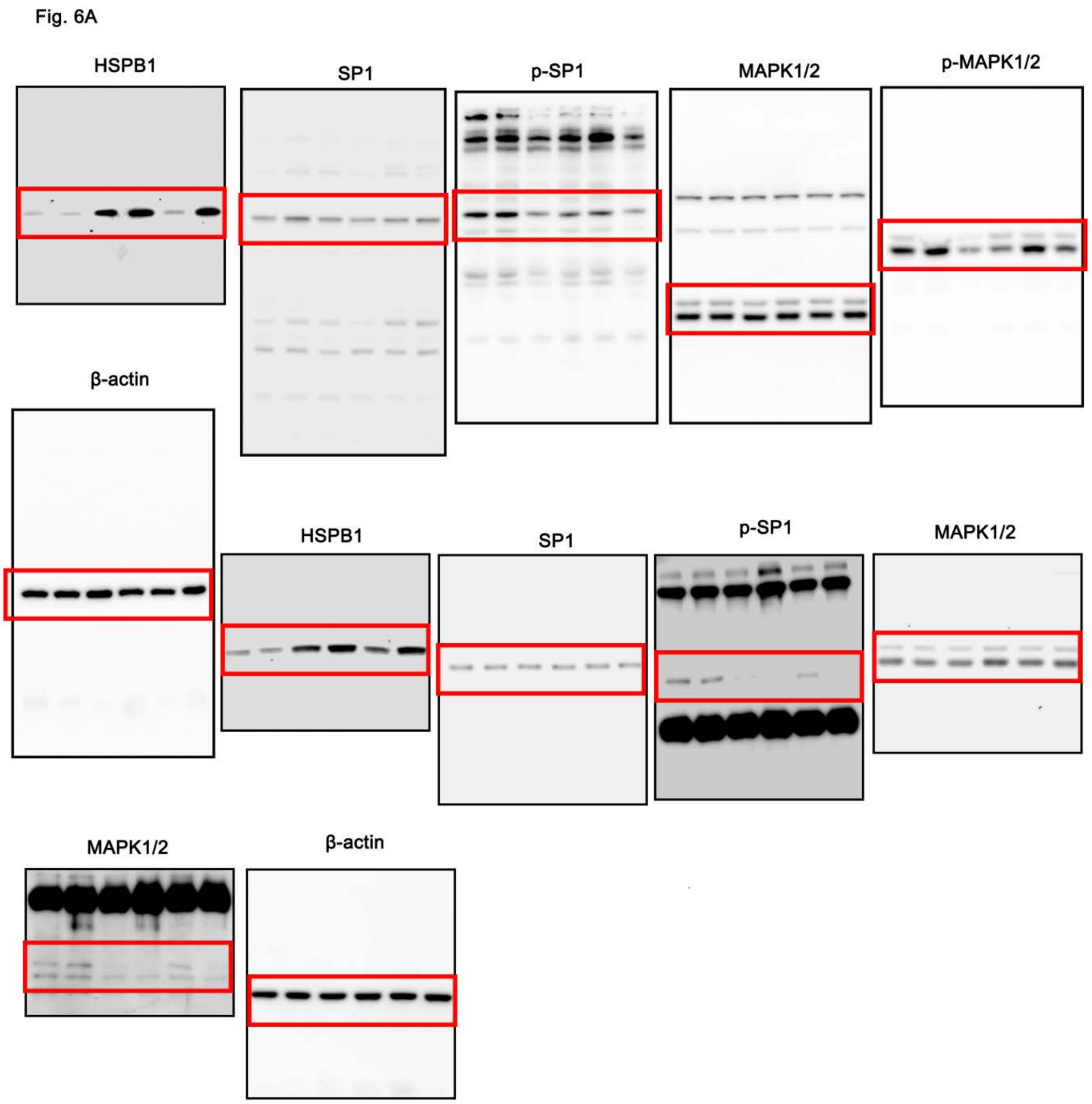

Supplementary Figure 19. Full-length gel images of western blot data in Fig. 6A. The cropped parts of western blots are indicated with boxes.

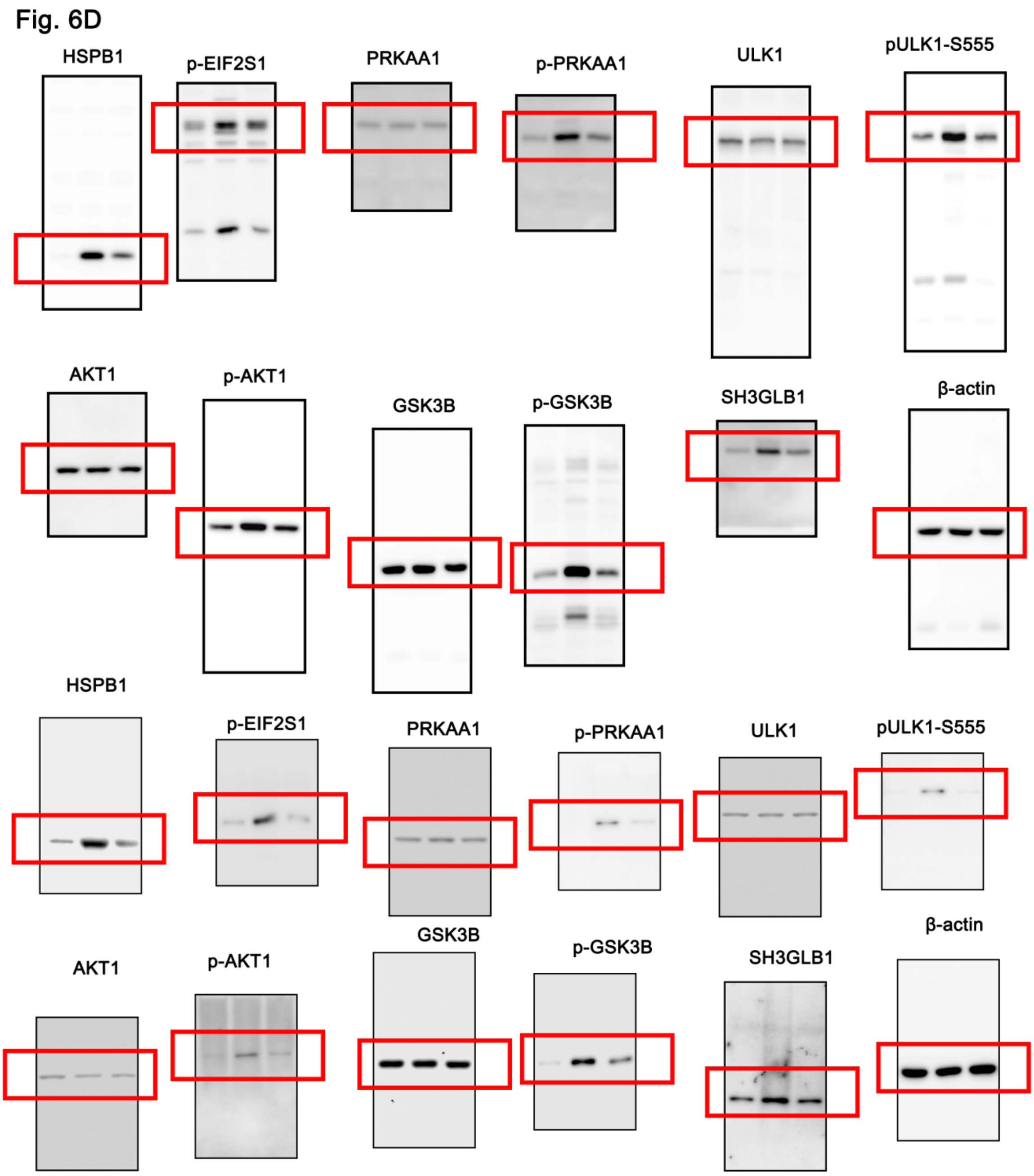

Supplementary Figure 20. Full-length gel images of western blot data in Fig. 6D. The cropped parts of western blots are indicated with boxes.

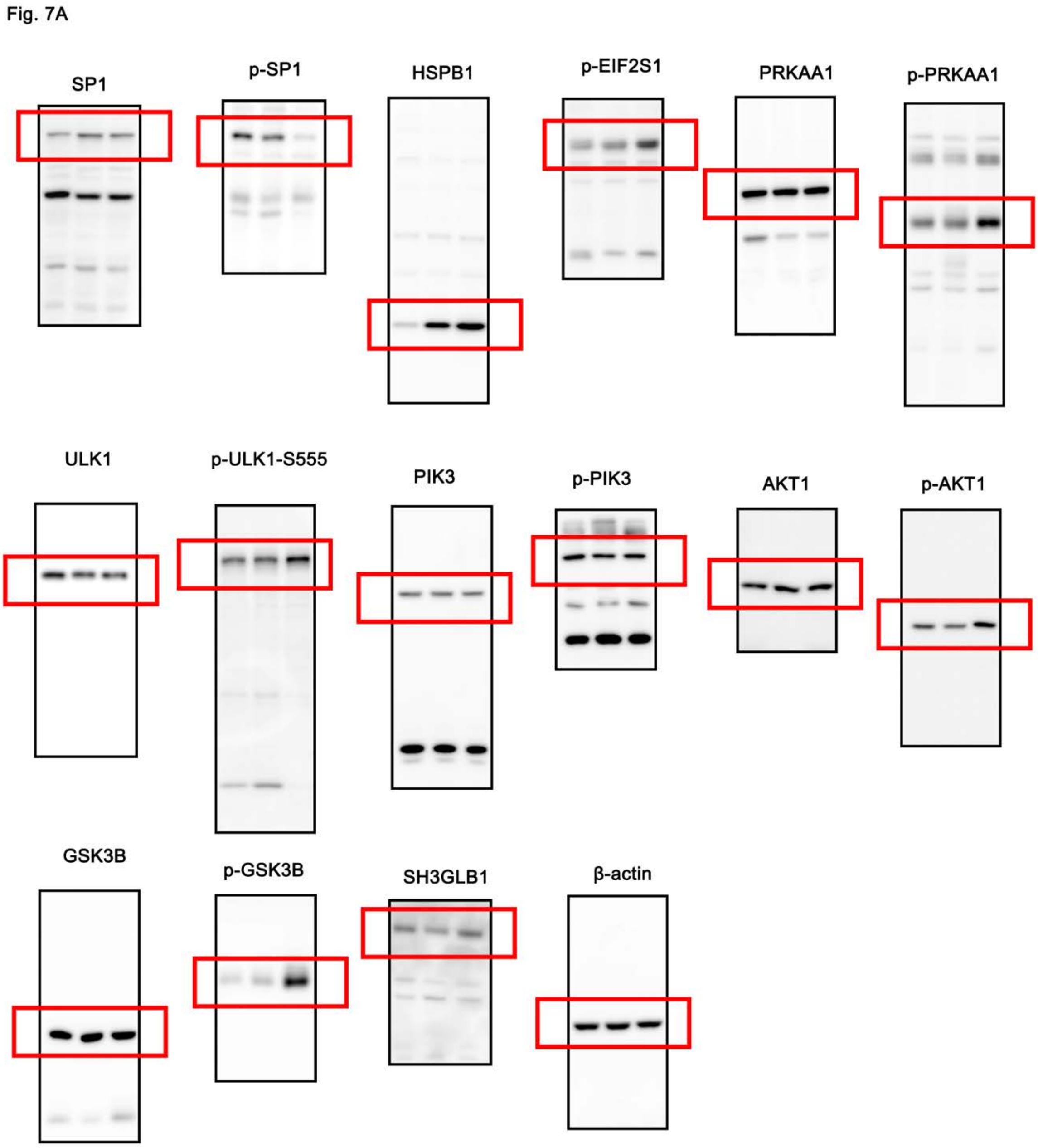

Supplementary Figure 21. Full-length gel images of western blot data in Fig. 7 in the whole hippocampus. The cropped parts of western blots are indicated with boxes.

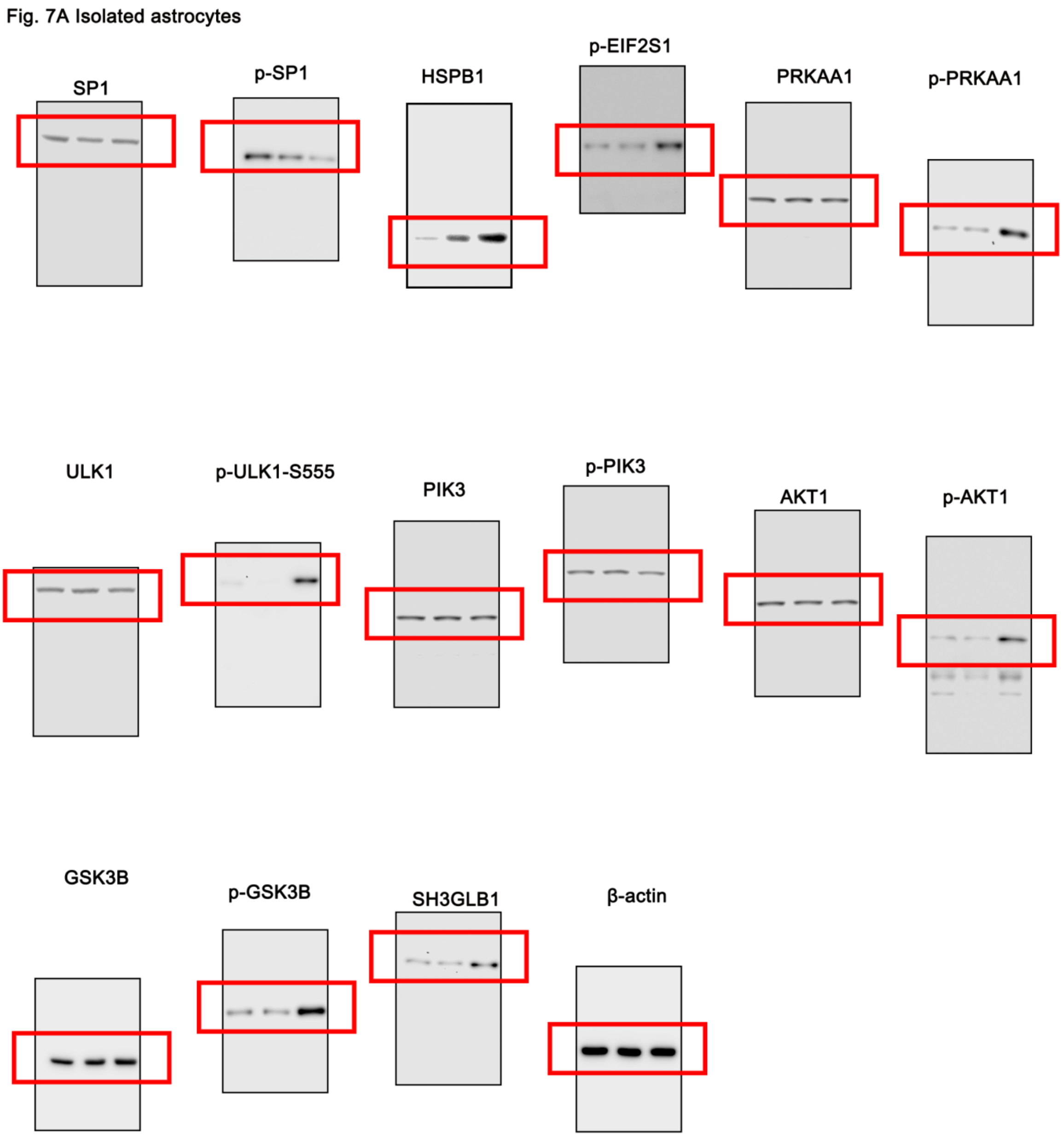

Supplementary Figure 22. Full-length gel images of western blot data in Fig. 7 in isolated astrocytes. The cropped parts of western blots are indicated with boxes.

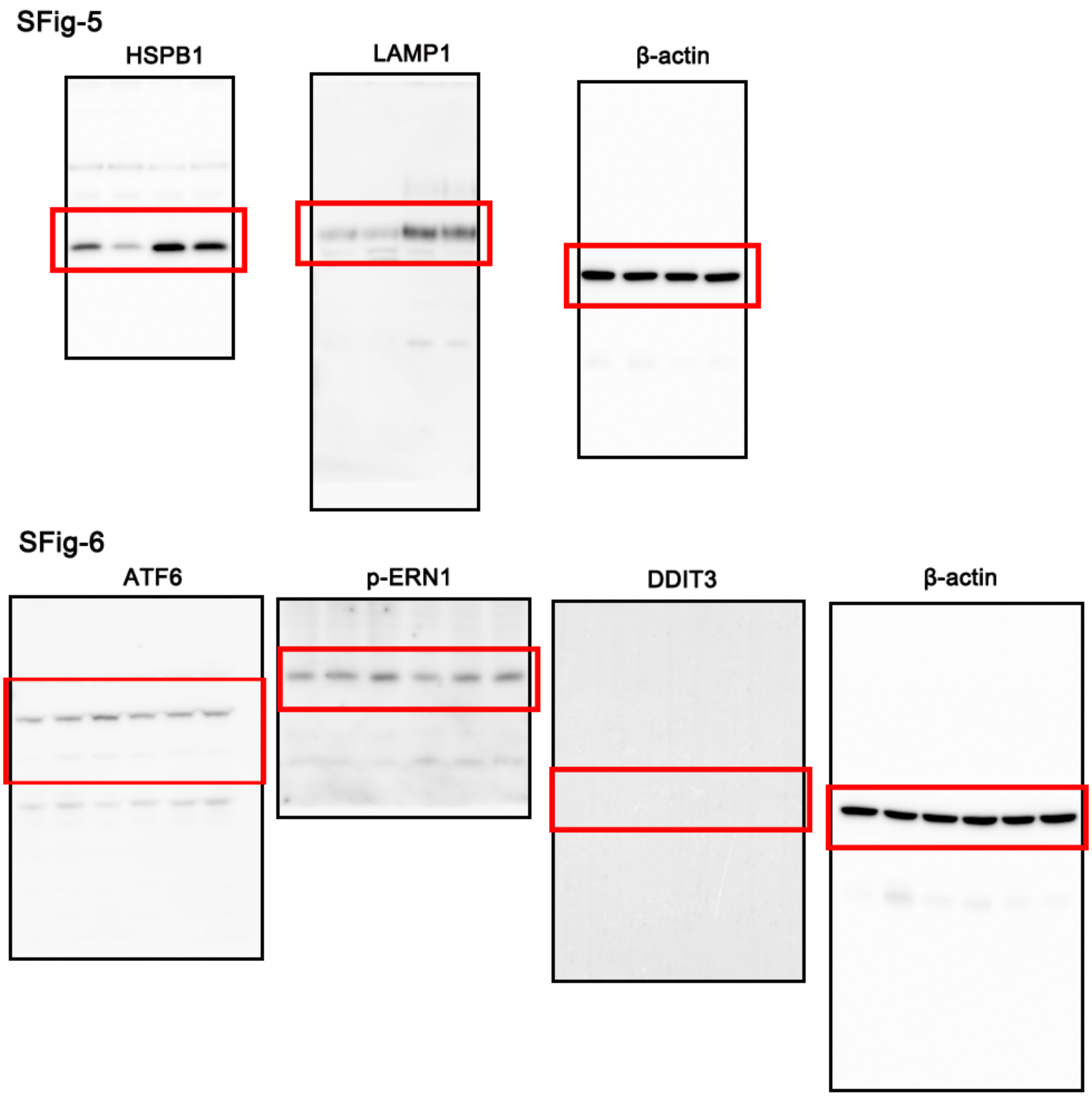

Supplementary Figure 23. Full-length gel images of western blot data in Supplementary Figs. 5-6. The cropped parts of western blots are indicated with boxes.

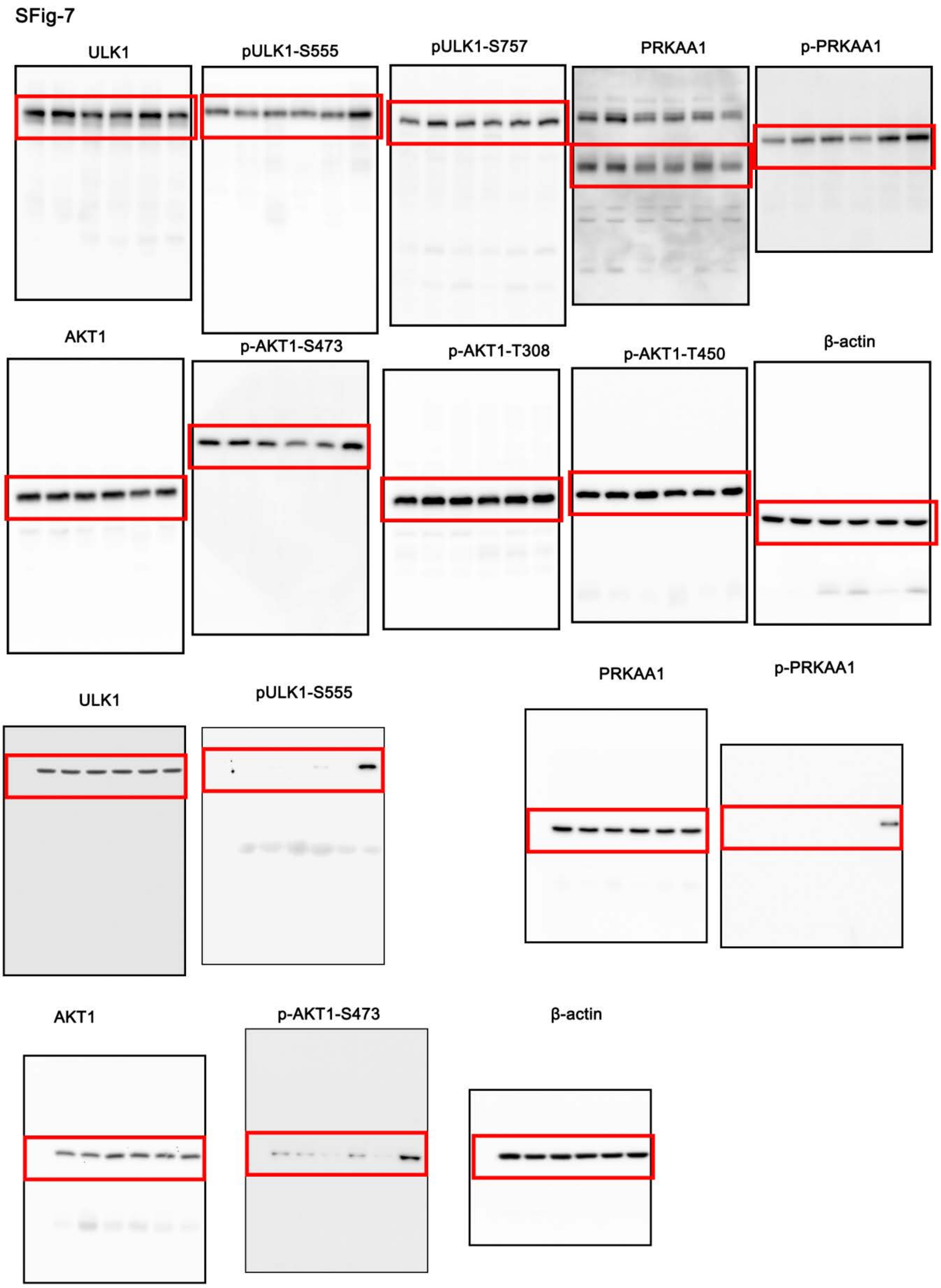

Supplementary Figure 24. Full-length gel images of western blot data in Supplementary Fig. 7. The cropped parts of western blots are indicated with boxes.

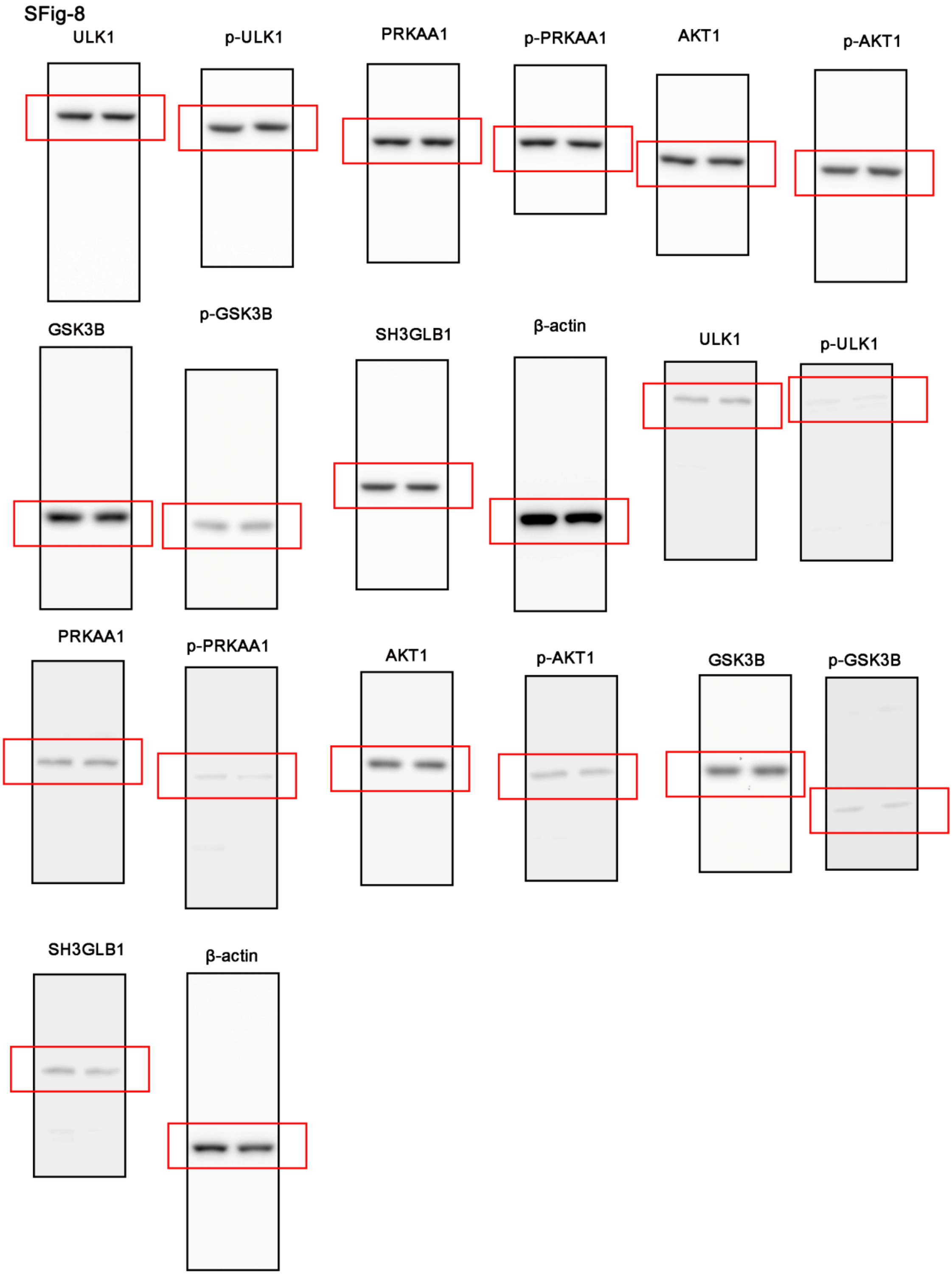

Supplementary Figure 25. Full-length gel images of western blot data in Supplementary Fig. 8. The cropped parts of western blots are indicated with boxes.

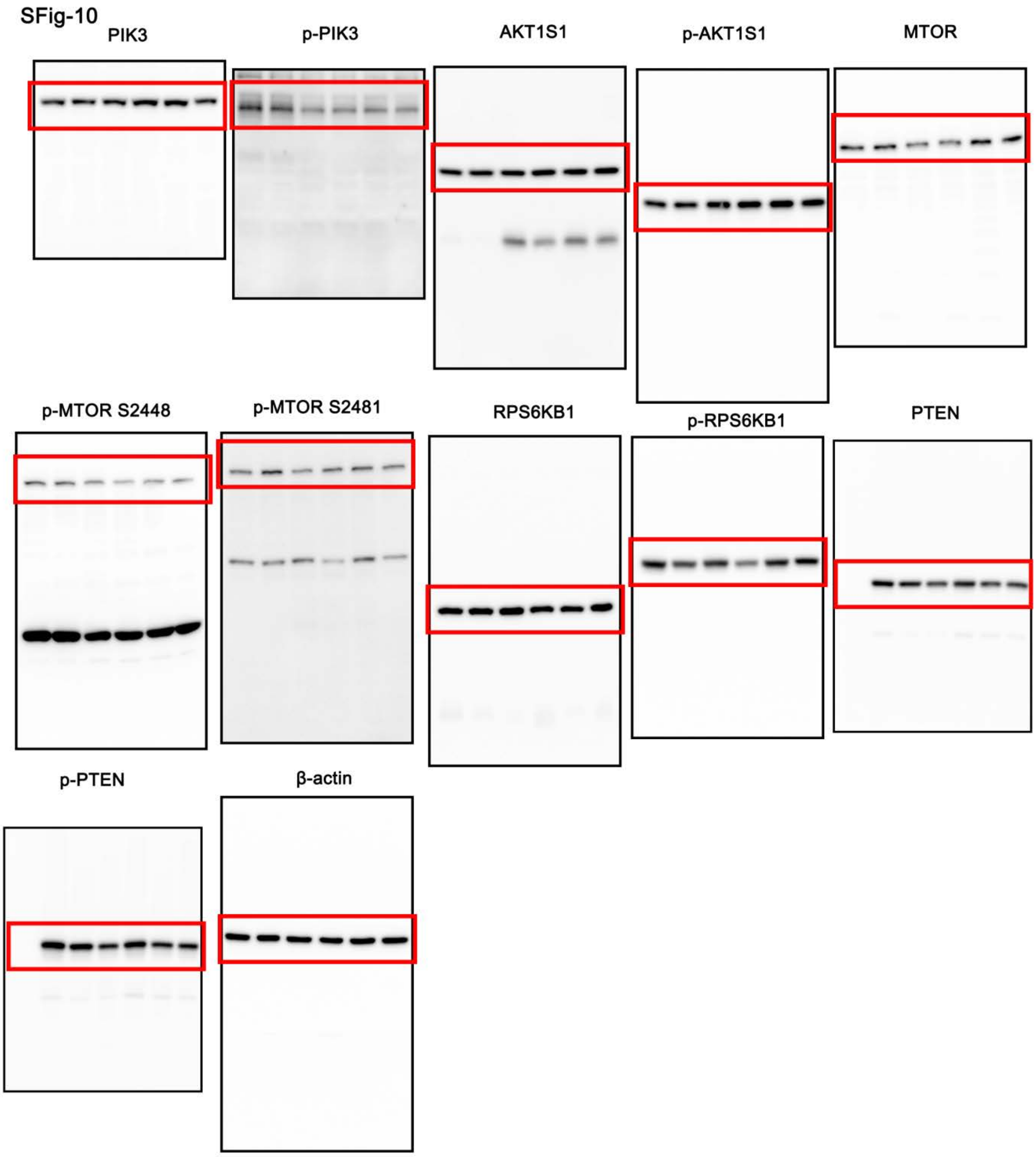

Supplementary Figure 26. Full-length gel images of western blot data in Supplementary Fig. 10. The cropped parts of western blots are indicated with boxes.

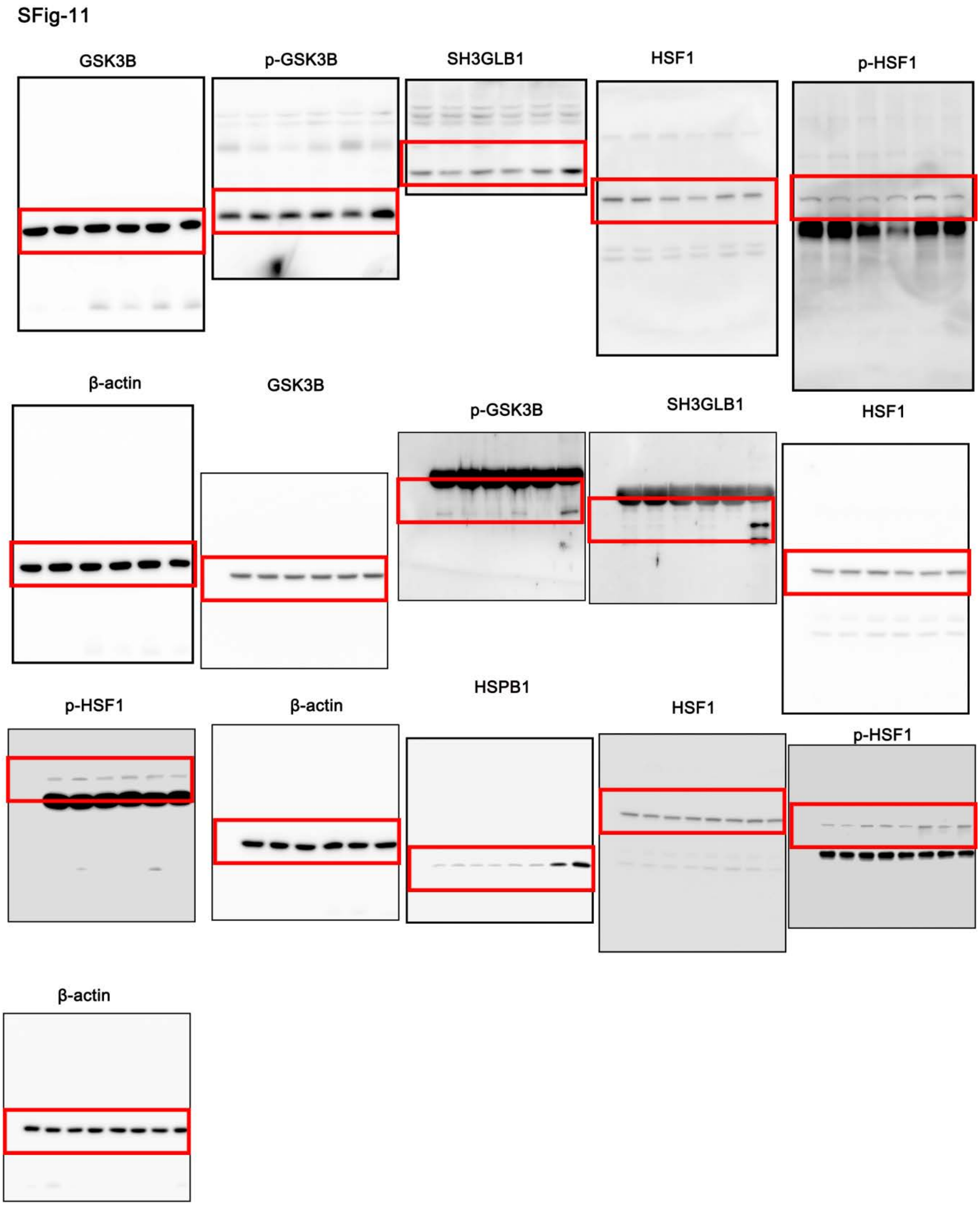

Supplementary Figure 27. Full-length gel images of western blot data in Supplementary Fig. 11. The cropped parts of western blots are indicated with boxes.
